# Temminck’s pangolins relax the precision of body temperature regulation when resources are scarce in a semi-arid environment

**DOI:** 10.1093/conphys/coad068

**Published:** 2023-08-28

**Authors:** Wendy Panaino, Francesca Parrini, Peter R Kamerman, Robyn S Hetem, Leith C R Meyer, Dylan Smith, Gus van Dyk, Andrea Fuller

**Affiliations:** School of Animal, Plant and Environmental Sciences, University of the Witwatersrand, Johannesburg, 2000, South Africa; Brain Function Research Group, School of Physiology, University of the Witwatersrand, Johannesburg, 2193, South Africa; School of Animal, Plant and Environmental Sciences, University of the Witwatersrand, Johannesburg, 2000, South Africa; Brain Function Research Group, School of Physiology, University of the Witwatersrand, Johannesburg, 2193, South Africa; School of Animal, Plant and Environmental Sciences, University of the Witwatersrand, Johannesburg, 2000, South Africa; Brain Function Research Group, School of Physiology, University of the Witwatersrand, Johannesburg, 2193, South Africa; Brain Function Research Group, School of Physiology, University of the Witwatersrand, Johannesburg, 2193, South Africa; Department of Paraclinical Sciences, and Centre for Veterinary Wildlife Research, University of Pretoria, Pretoria, 0110, South Africa; Tswalu Kalahari Reserve, van Zylsrus, 8467, Northern Cape, South Africa; Tswalu Kalahari Reserve, van Zylsrus, 8467, Northern Cape, South Africa; Brain Function Research Group, School of Physiology, University of the Witwatersrand, Johannesburg, 2193, South Africa; Department of Paraclinical Sciences, and Centre for Veterinary Wildlife Research, University of Pretoria, Pretoria, 0110, South Africa

**Keywords:** Kalahari, pangolins, thermoregulation

## Abstract

Climate change is impacting mammals both directly (for example, through increased heat) and indirectly (for example, through altered food resources). Understanding the physiological and behavioural responses of mammals in already hot and dry environments to fluctuations in the climate and food availability allows for a better understanding of how they will cope with a rapidly changing climate. We measured the body temperature of seven Temminck’s pangolins (*Smutsia temminckii*) in the semi-arid Kalahari for periods of between 4 months and 2 years. Pangolins regulated body temperature within a narrow range (34–36°C) over the 24-h cycle when food (and hence water, obtained from their prey) was abundant. When food resources were scarce, body temperature was regulated less precisely, 24-h minimum body temperatures were lower and the pangolins became more diurnally active, particularly during winter when prey was least available. The shift toward diurnal activity exposed pangolins to higher environmental heat loads, resulting in higher 24-h maximum body temperatures. Biologging of body temperature to detect heterothermy, or estimating food abundance (using pitfall trapping to monitor ant and termite availability), therefore provide tools to assess the welfare of this elusive but threatened mammal. Although the physiological and behavioural responses of pangolins buffered them against food scarcity during our study, whether this flexibility will be sufficient to allow them to cope with further reductions in food availability likely with climate change is unknown.

## Teaser

Pangolins need energy from food to maintain a constant and high body temperature, especially as they normally are active on cold nights. We found that when food was less available, pangolin body temperature was more variable, and pangolins emerged from their burrows earlier during the day.

## Introduction

Climate change is altering food availability, particularly in arid environments, through the links between altered precipitation, increased evapotranspiration and altered primary productivity, with cascading effects across trophic levels (Chesson *et al.,* 2004; Davis and Vincent, 2017; Fuller *et al.,* 2021). Mammals that are most likely to be threatened by changes in food availability associated with climate change are those with specialist diets, slow life histories and those that cannot readily move to more suitable habitats (Fuller *et al.,* 2016; Pacifici *et al.,* 2017). One such mammal is the Temminck’s pangolin (*Smutsia temminckii*), which is a predominantly nocturnal mammal with a myrmecophagous diet (specializing on a diet of ants and termites) (Pietersen *et al.,* 2016; Panaino *et al.,* 2022). Globally, pangolins are already threatened by habitat loss, electrocution on electrified fences, road mortalities and the illegal wildlife trade (Challender *et al.,* 2020).

Nocturnal mammals in arid zones, like the Temminck’s pangolin, must contend with low ambient temperatures and high radiative heat loss at night (Mitchell *et al.,* 2018; Fuller *et al.,* 2021). Maintenance of a relatively high body temperature at night requires a high metabolic rate, which in turn requires sufficient food energy (Jessen, 2001; Silva, 2005). When food becomes less available, nocturnal mammals may respond by reducing their metabolic rate and body temperature (Kelm and von Helversen, 2007; Fuller *et al.,* 2016; Hetem *et al.,* 2016), or by switching to diurnal activity (Van der Vinne *et al.,* 2019; Guiden and Orrock, 2020). During a drought in the Kalahari, aardvarks (*Orycteropus afer*), which also feed on ants and termites, had lower 24-h minimum body temperatures compared with non-drought years. They also emerged from their burrows more often during the daytime, sometimes becoming completely diurnal, and shortened their active periods by a quarter (Weyer *et al.,* 2020). Despite these responses, some aardvark still succumbed to starvation (Rey *et al.,* 2017; Weyer *et al.,* 2020). Switching to diurnal activity also exposes mammals to higher heat loads that they would otherwise avoid, potentially leading to hyperthermia, particularly if they have insufficient body water for evaporative cooling (Weyer *et al.,* 2020).

Temminck’s pangolins occur in the same environment as aardvarks and have a similar diet (Weyer, 2018; Panaino *et al.,* 2022), but their shallower foraging habits may make them vulnerable to fluctuations in food availability. Little is known about how Temminck’s pangolins respond to changing environments, and how they regulate body temperature and their activity patterns. Given that 24-h body temperature variability may be used to assess the wellbeing of free-living mammals (Hetem *et al.,* 2016; Maloney *et al.,* 2017), long-term pangolin body temperature data, combined with activity data, may provide insights into how these myrmecophagous mammals respond to seasonal and interannual fluctuations in climate and food resources, and, hence, the threat of climate change to their survival.

We therefore measured the core body temperature patterns of free-ranging pangolins in a semi-arid environment, to identify environmental factors associated with pangolin 24-h body temperature variability, to assess the relationship between behavioural responses of pangolins and 24-h body temperature variability and to assess the physiological consequences of those behavioural responses. We hypothesized that pangolins would regulate body temperature within a narrow 24-h range when food resources were adequate. When food resources were scarce, we predicted that pangolins would exhibit increased heterothermy as a result of decreased 24-h minimum body temperatures, and possibly also increased 24-h maximum body temperatures given that they obtain water primarily from their diet (Pietersen *et al.,* 2020). Because food energy is required to maintain constant high body temperatures, and some nocturnal mammals can shift activity to the day to offset metabolic costs of keeping warm at night, we predicted that the 24-h body temperature patterns of pangolins would also be related to their 24-h activity. We expected pangolins to increase diurnal activity when food was scarce, but that the increased diurnal activity would result in increased 24-h maximum body temperatures.

## Materials and Methods

### Study area

The study took place at Tswalu Kalahari Reserve (27°13’S, 22°26′E), situated in the southern Kalahari region of South Africa, within a semi-arid savannah biome; mean annual rainfall at Tswalu is 360 ± 170 mm (Tokura *et al.,* 2018). Typically, rainfall occurs in summer (December to February), with most rainfall events occurring in December and January, and winters (June to August) are dry. Air temperature ranges from ~−6°C in winter to 41°C in summer (onsite weather station, Vital Weather, CW Price & Co. (Pty) Ltd; period 2010–2017). Five vegetation types make up the reserve (Tokura *et al.,* 2018) but the study pangolins occurred predominantly in the Gordonia Duneveld and Gordonia Plains Shrubveld. Other myrmecophagous mammals inhabiting Tswalu include the aardvark, aardwolf (*Proteles cristata*) and bat-eared fox (*Otocyon megalotis*). Predators, including cheetahs (*Acinonyx jubatus*), African wild dogs (*Lycaon pictus*) and leopards (*Panthera pardus),* were present in our study area.

### Study animals and telemetry

We opportunistically located ten free-living adult pangolins (five males and five females, 6.5–9.4 kg) between April 2015 and March 2017. Once located, each pangolin was equipped with a Very High Frequency (VHF) tracking transmitter (~90 g, Africa Wildlife Tracking, Pretoria, South Africa) at the capture site. Pangolins typically curled into a ball when handled, making the attachment of tracking transmitters possible without the use of anaesthetic drugs. Two 3-mm holes were drilled into the non-vascularized portion of a single dorsolateral scale for attachment of the transmitter with two 3-mm bolts. Before drilling the holes, a strip of a material belt was placed under the scale to prevent injury to the animal while drilling. After securing the tracking transmitter (~10 min), the pangolin was released and allowed to roam freely. Contact was lost with three pangolins (one male and two females) subsequent to release. Therefore, seven pangolins (four males and three females) were used in our study.

### Body temperature measurement

A few months after the attachment of tracking transmitters, each pangolin was recaptured and underwent a small surgical procedure (see below) for the implantation of a miniature temperature-sensitive data logger (DST milli-T, Star Oddi, Garðabær, Iceland), which recorded core body temperature. The difficulty in finding pangolins initially meant that they were located, tagged with tracking transmitters, and underwent surgery on different dates, resulting in variability in the start and duration of body temperature logging (Supplementary Material, Fig. S1). Before implantation, each data logger was launched and set to record at 5-min intervals (Mercury v4, Star-Oddi, Garðabær, Iceland), then coated in inert wax (Sasolwax 1276, Sasol (Pty) Ltd, Sandton, South Africa). We calibrated the waxed data loggers (mass ± 20 g) before implantation and after being explanted from animals (see below) against a highly accurate thermometer (Quat 100, Heraeus, Germany) in a water bath over a range of 26–42°C. The data loggers drifted in time by ±1 min per month; therefore, appropriate time shift corrections were made before data analysis.

On the day of surgery, we captured (picked up) each pangolin during its active phase and placed it into a Perspex box (550 × 400 × 300 mm). A veterinarian experienced with data logger implants into wild mammals administered gaseous anaesthesia (8% isoflurane in oxygen, ISOFOR, SafeLine Pharmaceuticals (Pty) Ltd, Johannesburg, South Africa) through a 15-mm hole in the Perspex box. Once the pangolin was unconscious, we sexed it by examining the external genitalia, and weighed it in the Perspex box (MICRO SW 30, 40-kg capacity, 1-g precision, Associated Scale Corporation, Johannesburg, South Africa), subtracting the mass of the box afterwards. The pangolin was removed from the box and anaesthesia was maintained using isoflurane in oxygen (0.5–2% as needed) via a face mask.

We cleaned a 100 × 100 mm patch on the abdomen of the pangolin and sterilized the area using antiseptic solutions (Hibitane, chlorhexidine: 5%, and Hibicol, chlorhexidine gluconate: 0.5% in spirits, F10 Health and Hygiene (Pty) Ltd, Roodepoort, South Africa). A local anaesthetic (lignocaine hydrochloride, 20 mg/ml, 1–2 ml, Animal Health Division, Bayer HealthCare (Pty) Ltd, Kempton Park, South Africa) was injected subcutaneously into the sterilized site on the lower abdomen along the *linea alba* where the incision was to be made. A 15-mm incision was made, and the temperature data logger was inserted retroperitoneally and tethered to the midline with 2/0 non-absorbent Nylon suture material (Scimitar Surgical Sutures, Gabler Medical (Pty) Ltd, Essex, UK) to prevent the logger from drifting within the body of the animal. Further details about the surgery are included in the Supplementary material under *Surgical procedures.*

Immobilization was reversed by removing the face mask and allowing the pangolin to breathe natural air. Each pangolin was returned to the capture site, where an observer and a veterinarian monitored it until it walked with a steady gait. At the end of the data collection periods, pangolins were captured again, and data loggers were explanted. The methods used to explant data loggers from the pangolins were similar to those used to implant them. The implant sites had healed well with no signs of infection. The VHF tracking transmitters were left on the pangolins for at least 1 week after the explant surgeries before removal, so that we could track the animals to ensure that they had recovered fully. All procedures in our study followed animal ethics standards (University of the Witwatersrand Animal Research Ethics Committee, approval number 2015/04/16B). The relevant provincial permits were obtained for the work conducted in our study: FAUNA 1075/2015, FAUNA 1076/2015, FAUNA 1345/2017, FAUNA 1346/2017.

### Climatic variables

Black globe temperature was measured every 30 min by mounting a temperature-sensitive probe (HOBO Temperature/Relative Humidity/2 External Channel Data Logger, Onset Computer Corporation, Massachusetts, USA) into a 150-mm matt black globe. The globe was located at the Dedeben Research Centre, ~10 km from the area where study pangolins ranged. We obtained rainfall data for the study period from 32 rain gauges spread across Tswalu, covering the broad range of the pangolins. Times of sunrise and sunset were obtained from the South African Weather Services, and photoperiod was determined by calculating the time difference between sunrise and sunset. Seasons were defined as: summer (December to February), autumn (March to May), winter (June to August) and spring (September to November).

### Prey abundance

Prey abundance was recorded monthly using 300 pitfall traps (50-ml Falcon® Centrifuge Tubes, 27-mm diameter, Corning Inc., Massachusetts, USA) across 30 permanent transects (Pietersen, 2013). Each trap tube contained 20–30 ml antifreeze and water solution (1:10 commercial glycerol-based antifreeze-to-water ratio) and transects consisted of 10 tubes placed 5 m apart. Traps were placed in the ground with the opening of the trap level with the ground surface and remained in the ground for 4 days. Because ants dominated the pangolin diet (Panaino *et al.,* 2022) and termites rarely fell into the traps, only the ants that fell into the traps were counted and used as a proxy for pangolin prey abundance.

### Pangolin activity

Pangolins were tracked during their inactive phase using telemetry to locate their burrows. Camera traps (MMS wireless scouting camera, LTL-6210MC HD series, Ltl Acorn, Shenzhen, China) were placed outside the burrows to assess time of emergence and return of the pangolins. The camera traps did not always trigger for emergence and return events, and pangolins did not always return to the same burrow, thus the camera traps did not always detect time of emergence and return. However, inspection of body temperature records, for which we had observations of emergence and return to burrows, showed a conspicuous change (notch) in body temperature around time of emergence and return from and to the burrow (Supplementary Material, Fig. S2). We plotted each 24-h body temperature pattern for each pangolin to identify possible time of emergence and return, indicated by an increase or decrease in body temperature by at least 0.5°C in <1 h. We validated this method (Supplementary material, *Validation of pangolin activity*, Supplementary Material, Fig. S3) to estimate times of emergence and return to burrows and then supplemented the actual observations of emergence and return (from the camera traps) with estimated times of emergence and return derived from the 24-h body temperature patterns. Where there was no conspicuous body temperature change, time of emergence or return was not estimated. By combining camera trap data with activity data extracted from 24-h body temperature patterns, 2738 observations for time of emergence from the burrow and 2503 observations for time of return to the burrow were obtained for the study period.

### Data analyses

Data analyses were conducted using GraphPad Prism 8 (GraphPad, Massachusetts, USA) and R version 4.2.0 software (R Foundation, Vienna, Austria). For seasonal and yearly comparisons only, data were divided into four seasons (summer: December to February, autumn: March to May, winter: June to August and spring: September to October), and two year-long periods (November 2015 to October 2016: year 1, and November 2016 to October 2017: year 2). Because each year-long period started in November, dividing spring across 2 years, November was excluded from the calculation of springtime summaries. Thus, springtime summaries only consisted of 2 months of data (September and October).

#### Climatic variables

The 24-h mean, minimum, maximum and amplitude of black globe temperature were determined across the two study years, and for each season. Because of weather station failure, only 8 days of data were available for the autumn 2015/2016 (Supplementary Material, Fig. S4, Table S1), and hence autumnal data were excluded from the analyses. To assess seasonal (summer, winter, spring only) and yearly (excluding autumnal periods) patterns of 24-h black globe temperatures, linear regression models were run with period and season as main effects. All models were assessed for linearity (residual vs fitted plot), heteroskedasticity (residual vs fitted plot), outliers (Cook D > 1), normality of residuals (QQ-plot) and multicollinearity (variance inflation factor >5) (R package: ‘performance’, Lüdecke *et al.,* 2021). The average monthly rainfall was calculated by averaging rainfall across the rain gauges, and annual rainfall was calculated by summing the monthly averages.

#### Prey abundance

To assess seasonal and yearly differences in prey abundance (ant count per trap), a generalized linear mixed-effects model with a negative binomial link for over-dispersion was run, including season and year as fixed effects, and transect number as a random effect (R package: ‘lme4’, Kuznetsova *et al.,* 2017). Residual diagnostics were performed to check for correct distribution, outliers, over-dispersion and zero inflation (R package: ‘DHARMa’, Hartig, 2022).

#### Pangolin body temperature

To test for seasonal and yearly differences in pangolin 24-h mean, minimum, maximum and amplitude in body temperature, linear mixed-effects models were run, including season and year as fixed effects, and individual pangolins as a random effect (R package: ‘lme4’, Kuznetsova *et al.,* 2017), with 24-h periods starting at midnight. All models were assessed for linearity (residual vs fitted plot), heteroskedasticity (residual vs fitted plot), outliers (Cook D > 1), normality of residuals (QQ-plot) and multicollinearity (variance inflation factor >5) (R package: ‘performance’, Lüdecke *et al.,* 2021).

#### Associations between environmental conditions and pangolin body temperature

Linear mixed-effects models were used to the test the effects of photoperiod (day length), prey abundance (with the assumption that prey abundance was consistent for the month) and black globe temperatures on the 24-h minimum body temperatures, and on the amplitude of the 24-h rhythm of body temperature, with individual pangolins as a random effect. When modelling relationships with 24-h maximum body temperature, an additional variable, burrow emergence time, was included in the model. Exploratory plots indicated that the relationship between prey abundance and the three body temperature variables showed an inflection point (visually, we estimated that it would be somewhere between 0 and 18 ants per trap); therefore, a piecewise regression (R package: ‘lspline’, Perperoglou *et al.,* 2019) was used to assess the relationship before and after a breakpoint. A search was conducted to determine the best breakpoint by running the models using various breakpoints between 0 and 18 ants per trap, and then comparing the models for the best fit using Akaike Information Criterion (AIC), where the model with the lowest AIC value is selected as the best model (Symonds and Moussalli, 2011). Because the time-of-day data are circular, burrow emergence times (included in the model for 24-h maximum body temperature only) were processed to only include times from 04:00 to 23:55 (Supplementary Material, Fig. S5, and Table S2). This processing resulted in a small loss of some data (Supplementary Material, Table S2), but it overcame the issue of the circular nature of the data (e.g. emergence times at 01:00 are only 2 h apart from 23:00 on the previous day, but 22 h apart from 23:00 on the same day). As we did for pangolin body temperature, all models were assessed for linearity, heteroskedasticity, outliers and multicollinearity. Where heteroskedasticity was detected, robust standard errors were calculated (R packages: ‘parameters’ and ‘sandwich’, Lüdecke *et al.,* 2020, Zeileis, 2006 and Zeileis *et al.,* 2020).

## Results

### Climatic variables

Black globe temperatures ranged from a minimum of −5.1°C (winter) to a maximum of 49.5°C (summer) in year 1, and from a minimum of −4.2°C (winter) to a maximum of 50.4°C (summer) in year 2. Across seasons, the 24-h minimum globe temperature was, on average, 4.0°C (SE: 0.3) lower in year 2 compared with year 1 (Supplementary Material, Tables S3 and S4, Fig. S6). The mean difference for 24-h maximum black globe temperatures between year 1 and year 2 was 0.7°C (SE: 0.4) (Supplementary Material, Tables S5 and S6, Fig. S7). Consistent with a lower 24-h minimum black globe temperature in year 2 compared with year 1, along with the small annual difference in mean 24-h maximum black globe temperature, the 24-h amplitude of black globe temperature was greater in year 2 compared with year 1 by 4.7°C (SE: 0.5) (Supplementary Material, Tables S7 and S8, Fig. S8). When averaged across seasons, the 24-h mean black globe temperature in year 2 was 2.1°C (SE: 0.3) greater compared with year 1 (Supplementary Material, Tables S9 and S10, Fig. S9). When averaged across years, 24-h mean, minimum and maximum black globe temperatures were greatest in summer and lowest in winter (Supplementary Material, Tables S4, S6, and S10). Daily amplitude of black globe temperatures did not, however, change across the seasons (Supplementary Material, Table S8).

Across the 2-year period, median (interquartile range [IQR]) rainfall was greatest in summer (37 mm [8–76]) and autumn (26 mm [12–33]) months, and lowest in spring (5 mm [0–12]) and winter months (0 mm [0–0]) (Supplementary Material, Table S11, Fig. S10, Fig. S11). Greater rainfall was recorded in year 2 (391 mm) compared with year 1 (217 mm) (Supplementary Material, Table S12, Fig. S12).

### Prey abundance

Monthly ant abundance varied appreciably across seasons and the year of study (Supplementary Material, Table S13 and S14, Fig. S13 and Fig. S14). Averaging across seasons, ant abundance was 56% lower during year 1 (mean: 11.4 ants/trap, SE: 1.2) compared with year 2 (mean: 20.4 ants/trap, SE: 2.2), and was lowest during autumn (mean: 9.9 ants/trap, SE: 1.3) and winter (mean: 8.2 ants/trap, SE: 1.1), and highest during summer (mean: 20.2 ants/trap, SE: 3.1) and spring (mean: 26.9 ants/trap, SE: 3.4).

### Pangolin body temperature

**Figure 1:**
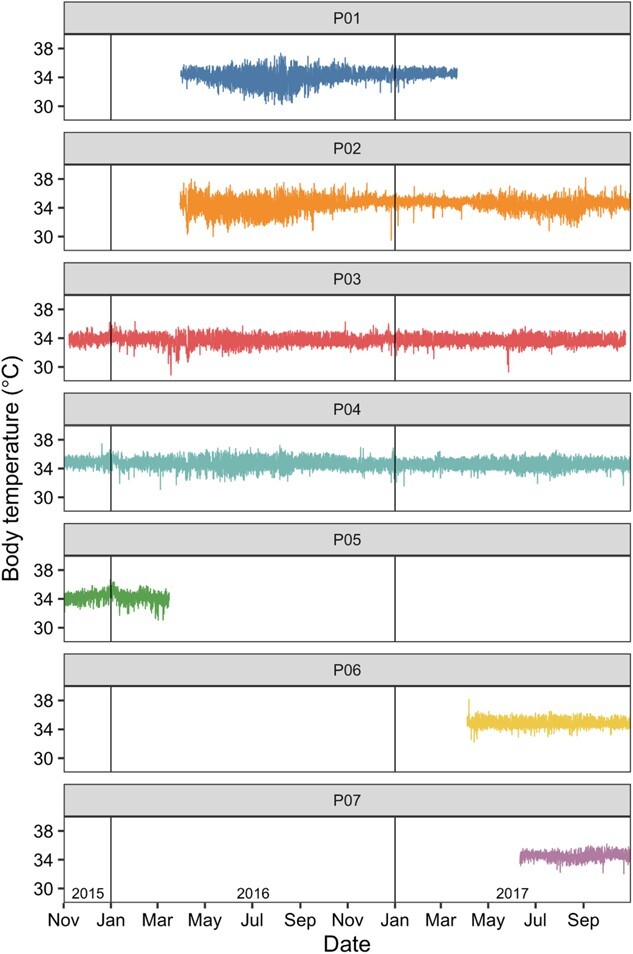
Body temperatures recorded at 5-min intervals for seven individual pangolins across the study period. P01 to P07 represent the different study animals.

Body temperature was recorded for seven pangolins for a total of 2938 pangolin days across the study period (November 2015 to October 2017; Fig. 1). Body temperature data showed a unimodal distribution whereby pangolins predominantly regulated body temperature between 34 and 36°C ~92% of the time. The absolute minimum 24-h body temperature recorded was 28.9°C (March 2016) and the absolute maximum 24-h body temperature recorded was 38.2°C (September 2017). The maximum amplitude of the 24-h rhythm of body temperature was 6.8°C, with 24-h body temperature increasing from a minimum of 30.2°C to a maximum of 37.0°C (within 2 h and 39 min) during July 2016.

When averaging across seasons, 24-h minimum body temperature was greater in year 2 than in year 1 (mean difference: 0.35°C, SE: 0.03). Seasonally, when averaged across years, summer was the season when pangolins had the highest 24-h minimum body temperature (mean: 33.7°C, SE: 0.17) and winter was when body temperature was lowest (mean: 32.9°C, SE: 0.17) (Supplementary Material, Tables S15 and S16, Fig. S15). Pangolin 24-h maximum body temperature, when averaged across season, was lower in year 2 than in year 1 (mean difference: 0.27°C, SE: 0.02), but there was no appreciable seasonal change in 24-h maximum body temperature (Supplementary Material, Tables S17 and S18, Fig. S16). Consistent with the patterns of 24-h maximum and 24-h minimum body temperatures between years, the 24-h amplitude of body temperature of pangolins was greater in year 2 than in year 1. When averaging across years, there was a seasonal pattern in the 24-h amplitude of body temperature, with pangolins having a smaller amplitude in summer than in winter (mean difference: 0.80°C, SE: 0.04) (Supplementary Material, Tables S19 and S20, Fig. S17). Mean 24-h body temperature, when averaged across seasons, was highest during summer and lowest during winter (mean difference: 0.34°C, SE: 0.01). There was little difference between yearly 24-h mean body temperatures (Supplementary Material, Tables S21 and S22, Fig. S18).

**Figure 2:**
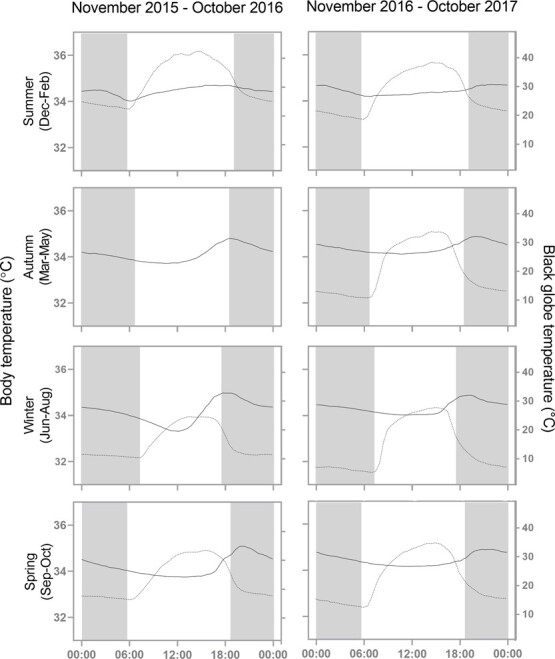
The seasonal and interannual 24-h body temperature patterns of pangolins for the two study years. The black lines represent the average body temperature through 24 h across all seven pangolins across each month in that season. The black dashed lines represent the average 24-h black globe temperatures averaged across each month in that season. Weather data for autumn 2016 are excluded due to weather station failure. The grey-shaded areas represent the dark phase.

Figure 2 shows the relationship between mean 24-h body temperature and mean 24-h black globe temperature across the study period (December 2015 to October 2017) for each season (summer, autumn, winter and spring).

**Figure 3:**
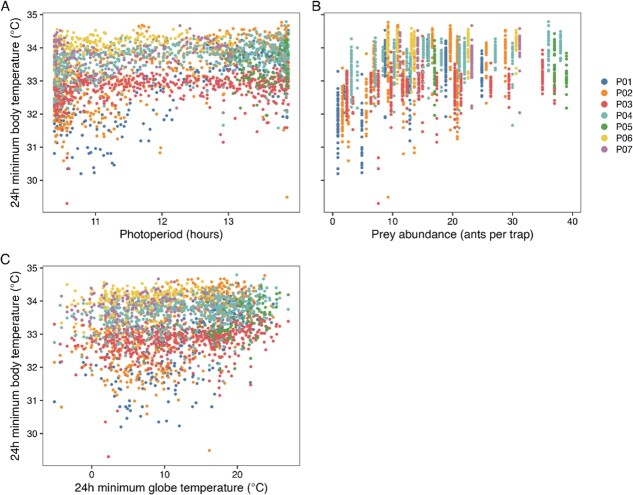
The relationship between pangolin 24-h minimum body temperature and photoperiod (A), prey abundance (B) and 24-h minimum black globe temperature (C). P01 to P07 represent the different study animals.

The linear mixed model analyses (Table 1) revealed that pangolin 24-h minimum body temperature was positively associated with photoperiod (Fig. 3A), prey abundance (Fig. 3B) and 24-h minimum black globe temperature (Fig. 3C). In other words, on shorter days (in winter), lower prey abundance (at abundance levels <9 ants/trap) and lower environmental temperatures were associated with lowering of pangolin 24-h minimum body temperature. Although all these associations were statistically significant, the relationships described by the model were consistent with small effect sizes. There was no statistically significant association between prey abundance and 24-h minimum body temperature at a prey abundance ≥9 ants per trap.

**Table 1:** Linear mixed model results of the effects of photoperiod, prey abundance and 24-h minimum black globe temperature on pangolin 24-h minimum body temperature

**Explanatory variable**	**Coefficient**	**SE**	**95% CI**	***t***	***P***
Intercept	31.30	0.28	30.75–31.85	112.28	<0.001
Photoperiod	0.09	0.02	0.05–0.13	4.42	<0.001
Prey abundance (<9 ants/trap)	0.10	<0.01	0.08–0.11	14.51	<0.001
Prey abundance (>9 ants/trap)	<−0.001	<0.01	0.00–0.00	−0.42	0.676
24-h minimum black globe temperature	0.02	<0.01	0.01–0.02	5.70	<0.001

**Figure 4:**
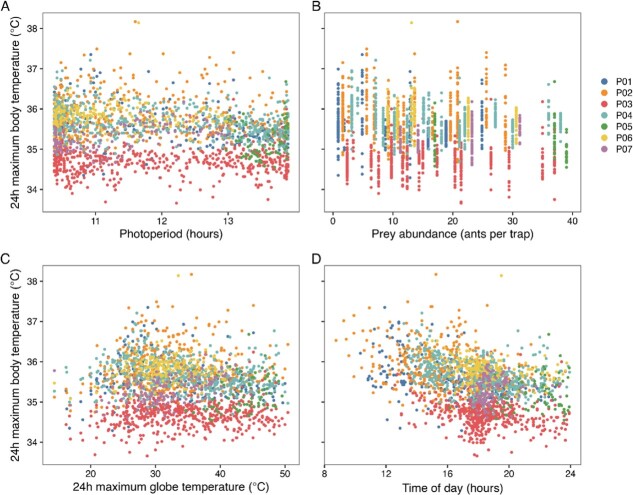
The relationship between 24-h maximum body temperature and photoperiod (A), prey abundance (B), 24-h maximum black globe temperature (C) and the time of emergence from the burrow (D). P01 to P07 represent the different study animals.

The linear mixed model analyses (Table 2) revealed that pangolin 24-h maximum body temperature was positively associated with photoperiod (Fig. 4A) and 24-h maximum black globe temperature (Fig. 4C), but was negatively associated with prey abundance (<10 ants/trap; Fig. 4B), and time of burrow emergence (Fig. 4D). In other words, pangolin 24-h maximum body temperature increased as days got longer, as 24-h maximum black globe temperature increased, as prey abundance decreased (at abundance levels <10 ants/trap) and as burrow emergence shifted to earlier in the day. Although all these associations were statistically significant, the relationships described by the model were consistent with small effect sizes. There was no statistically significant association between prey abundance and 24-h maximum body temperature at a prey abundance of ≥10 ants per trap.

**Table 2:** Linear mixed model results of the effects of photoperiod, prey abundance, 24-h maximum black globe temperature and time of emergence from the burrow on pangolin 24-h maximum body temperature

**Explanatory variable**	**Coefficient**	**SE**	**95% CI**	***t***	***P***
Intercept	36.18	0.18	35.83–36.54	112.28	<0.001
Photoperiod	0.05	0.01	0.03–0.08	4.42	<0.001
Prey abundance (<10 ants/trap)	−0.03	<0.01	−0.03 to −0.02	14.51	<0.001
Prey abundance (>10 ants/trap)	0.001	<0.01	0.00–0.00	−0.42	0.676
24-h maximum black globe temperature	0.03	<0.01	0.02–0.03	5.70	<0.001
Time of emergence from burrow	−0.11	<0.01	−0.12–−0.10	−19.36	<0.001

**Figure 5:**
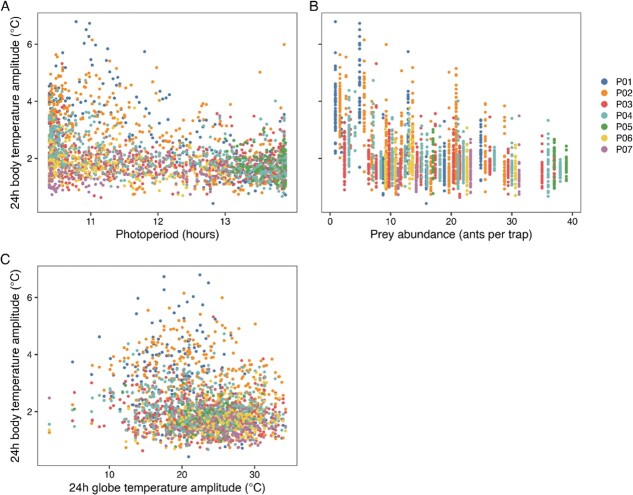
The relationship between amplitude of the 24-h rhythm of body temperature and photoperiod (A), prey abundance (B) and the amplitude of 24-h black globe temperature (C). P01 to P07 represent the different study animals.

The linear mixed model analyses (Table 3) revealed that the amplitude of the 24-h rhythm of pangolin body temperature was negatively associated with photoperiod (Fig. 5A) and a prey abundance <10 ants per trap (Fig. 5B). Although statistically significant, these relationships described by the model were consistent with weak effects sizes. There was no statistically significant association between the amplitude of the 24-h rhythm of black globe temperature (Fig. 5C) or a prey abundance of ≥10 ants per trap (Fig. 5B) and 24-h amplitude of body temperature. In other words, shorter days (in winter) and low prey abundance (at abundance levels <10 ants/trap) were associated with greater amplitudes of the 24-h rhythm of pangolin body temperature.

**Table 3:** Linear mixed model results of the effects of photoperiod, prey abundance and the amplitude of the 24-h rhythm of black globe temperature on the amplitude of the 24-h rhythm of pangolin body temperature

**Explanatory variable**	**Coefficient**	**SE**	**95% CI**	***t***	***P***
Intercept	5.29	1.26	2.82–7.77	4.19	<0.001
Photoperiod	−0.20	0.08	−0.35 to −0.04	−2.51	0.012
Prey abundance (<10 ants/trap)	−0.11	0.03	−0.17 to −0.04	−3.22	0.001
Prey abundance (>10 ants/trap)	<0.001	<0.01	−0.01–0.02	0.98	0.328
24-h black globe temperature amplitude	<0.001	<0.01	−0.01–0.01	0.07	0.942

## Discussion

Pangolins regulated body temperature within a narrow daily range (between 34 and 36°C) during summer. During winter, and during the year with less rainfall, which coincided with less abundant prey resources, they became more heterothermic, as indicated by decreased 24-h body temperature minima and increased 24-h body temperature maxima. Body temperatures of the pangolins fluctuated more through 24-h during the year in which prey resources were scarce compared with the year in which prey resources were abundant. The main predictors of pangolin body temperature parameters, including the 24-h minimum, maximum and amplitude of the 24-h rhythm of body temperature, were photoperiod and prey abundance. A shorter photoperiod, indicative of season, and a lower prey abundance were associated with a lower 24-h minimum body temperature, and a lower 24-h maximum body temperature, but the animals were more heterothermic. Pangolin body temperature was also associated with environmental heat load, with lower 24-h minimum and maximum body temperatures associated with lower 24-h minimum and 24-h maximum globe temperatures, respectively. However, the relationship between environmental temperatures and body temperature patterns was weak. In addition, lower 24-h minimum body temperature, higher 24-h maximum body temperature and lower prey abundance coincided with pangolins emerging from their burrows earlier during the day.

Although our study is the first to investigate the body temperature patterns of wild pangolins, we were able to collect data continuously from only two animals for the full 2 years because we lost contact with some pangolins. However, at any given time, body temperature data were collected from at least three pangolins, allowing us to have some confidence in detecting differences between the two study years. To reduce the chance of losing contact with these animals that move large distances, future studies should include satellite tags with the VHF tracking transmitters. Our small sample size did not allow us to elucidate any possible differences based on sex or body size. We also detected small effect sizes in some of our models. Nevertheless, we present the most comprehensive data on body temperature collected to date for this elusive mammal, which reveal important insights for this species.

The body temperature of pangolins has been found to be lower than that of other placental mammals of similar size, likely reflecting their low metabolic rate (McNab, 1984; Griebeler and Werner, 2016; Boyles *et al.,* 2019). Pangolins in our study, however, had mean body temperatures that were higher compared with those measured previously for Temminck’s pangolins in captivity (32–34°C; Boyles *et al.,* 2019; Wicker *et al.,* 2020) and for one animal in the wild (32–35°C; Pietersen, 2013). The highest (absolute maximum) body temperature (38.2°C) we measured was higher than the maximum body temperatures reported previously for any pangolin species in captivity (33.9–37.8°C, Jones, 1973; McNab, 1984; Heath and Hammel, 1986; Heath, 1987; Boyles *et al.,* 2019; Challender *et al.,* 2020; Yu *et al.,* 2021) and almost 3°C higher than that previously reported for a wild Temminck’s pangolin (35.4°C), measured across 34 days in winter (Pietersen, 2013). The lowest body temperature recorded for a pangolin in our study (28.9°C in March 2016) was within the range of minimum body temperatures recorded previously for captive animals (~27.6–33.4°C; McNab, 1984; Heath and Hammel, 1986; Heath, 1987; Yu *et al.,* 2021) and similar to that recorded for a wild pangolin (29.5°C; Pietersen, 2013). Although data on the body temperature of pangolins in captivity are useful for understanding pangolin physiology, the data likely do not accurately reflect the body temperature patterns of wild pangolins exposed to natural conditions. Pangolins housed under captive conditions are not as active as wild pangolins, may be in poor condition and likely are not exposed to high radiant heat loads like the pangolins in our study were when they emerged from their burrows diurnally, which may explain the higher maximum body temperature recorded in our study. However, the body condition and nutritional status of pangolins in captivity have not been reported.

The main reason we measured body temperature in pangolins was to investigate whether patterns of body temperature were related to changing food availability. Food energy is required for mammals to generate the metabolic heat needed to maintain relatively high and constant body temperatures (Hetem *et al.,* 2016). Pangolins maintained homeothermy (between 34 and 36°C) throughout most of the year, but they exhibited heterothermy during winter. Pangolin 24-h minimum body temperature was closely linked to prey abundance, such that when prey abundance was low (<9 ants/trap) during winter, body temperature fell more than normal. Low 24-h minimum body temperatures presumably allowed pangolins to conserve energy by reducing metabolism, as explained by the Q10 effect in mammals (Heldmaier and Ruf, 1992). Another advantage of the resulting lower 24-h minimum body temperatures is that the temperature difference between the animal’s body surface and the environment would be lower, thereby reducing dry heat loss to the environment, and reducing the energy demand for heat production. By relaxing the precision of body temperature regulation during winter, pangolins, like many other mammals (Pereira *et al.,* 2002; Hetem *et al.,* 2016; Theimer *et al.,* 2017; Weyer *et al.,* 2020), seemed to prioritize energy conservation over thermoregulation during a period when food was scarce. Low body temperatures, however, may put mammals at risk of hypothermia and death, as seen in starving aardvarks (Rey *et al.,* 2017; Weyer *et al.,* 2020). Pangolins in our study had 24-h mean body temperatures as low as 32°C in the year with food scarcity, ~1°C lower than in the year with available food. The dry year during our study was not as extreme as had been experienced previously in the Kalahari (such as the year 2013, for example, Rey *et al.,* 2017). Therefore, whether pangolins may be able to tolerate even lower body temperatures in drought years, as a buffer against food scarcity, or in the future as climate change reduces resource availability further, is unknown.

Low 24-h minimum body temperatures during winter when food was scarce coincided with pangolins becoming active earlier during the day. Pangolins shifted from a predominantly nocturnal pattern during summer to greater diurnal activity during winter, presumably to reduce the metabolic costs of staying warm at night. Even during summer, the effective temperature of the night sky in drylands is between −10 and − 30°C, making the gradient for radiant heat loss substantial (Mitchell *et al.,* 2018). In winter, low air temperatures facilitate even greater dry heat loss (Fuller *et al.,* 2021). Therefore, a shift in 24-h activity between summer and winter likely allowed pangolins to conserve energy by reducing potential heat loss, as has been shown for nocturnal white-footed mice (*Peromyscus leucopus*), where a shift in 24-h activity during winter resulted in a reduced potential heat loss of 4% (Guiden and Orrock, 2020).

A shift to diurnal activity does not necessarily come without costs. Even during winter, black globe temperatures can be as high as 37°C in the late afternoon in the Kalahari. Pangolins are poorly insulated by their scale covering (Heath and Hammel, 1986; Weber *et al.,* 1986; Challender *et al.,* 2020). The poor insulation, together with their relatively small body size, means that they would likely gain heat rapidly when exposed to heat loads. Indeed, when pangolins emerged from their burrows in the afternoon, they exhibited increased 24-h maximum body temperatures after the emergence. The rapid rise in body temperature is unlikely to reflect an increase in metabolism, given that the pangolins lack sufficient food energy. Pangolins also lack UCP1 (Gaudry *et al.,* 2018) and therefore cannot implement non-shivering thermogenesis. It is more likely that direct exposure to high heat loads during the day were responsible for the increased 24-h maximum body temperatures.

Although food energy is important for the maintenance of high body temperatures, sufficient body water is needed for evaporative cooling (Ostrowski *et al.,* 2003; Fuller *et al.,* 2016; Hetem *et al.,* 2016). Evaporative cooling is the only way to lose heat when operative temperature exceeds an animal’s surface temperature (Mitchell *et al.,* 2018). Pangolins rarely actively seek freestanding water and obtain almost all their body water through their diet (Pietersen *et al.,* 2020). At our study site, water holes were present, but in 7 years of being in the field there studying pangolins for various purposes, we have never observed one drinking from these sources. On one occasion, we observed a pangolin drinking from a cup-like woody structure at the base of a shrub that had filled with water after a rain event during summer, suggesting that they will opportunistically drink water at times. Because pangolins get most of their body water from their food, when they experienced energy deficits as a consequence of reduced prey abundance, they likely also experienced water deficits. Indeed, pangolins exhibited rapid increases in body temperature during winter when prey abundance was low (<10 ants/trap) and when they emerged before nightfall, suggesting that they may have had insufficient body water to prevent the rise in body temperature. In mammals, dehydration reduces the rate of evaporative cooling or the threshold temperature at which evaporative cooling is activated (Fuller *et al.,* 2016). Pangolins lack sweat glands (Hyman and Wake, 1992), and it is unknown if cutaneous evaporation can occur across the skin, as it does, for example, in elephants (Dunkin *et al.,* 2013). It is also not clear if they lose heat though panting, but an increased breathing rate was observed in Chinese pangolins (*Manis pentadactyla*) when air temperatures rose >32°C (Heath and Hammel, 1986).

The amplitude of the 24-h rhythm of body temperature has been proposed as a proxy for the physiological welfare of mammals (Hetem *et al.,* 2016; Maloney *et al.,* 2017; Maloney *et al.,* 2019), with many mammals exhibiting homeothermy only when they have sufficient food and water and are not energetically challenged. Our pangolins exhibited amplitudes of the 24-h rhythm of body temperature that were inversely related to prey abundance, such that when prey abundance, and hence food energy and water, was low, pangolins exhibited greater amplitudes of the 24-h rhythm of body temperature compared with when prey was abundant. Similar findings were reported for aardvarks in the Kalahari; when drought conditions reduced grass, the availability of termites (*Hodotermes mossambicus*) decreased (Weyer, 2018), and aardvarks, which also obtain water from food, exhibited greater amplitudes of the 24-h rhythm of body temperature (Weyer *et al.,* 2020). At the same time, the aardvarks’ body condition deteriorated, and some succumbed. Although pangolin body condition was not measured in our study and could not be visually observed given the scale covering on the animals, Pietersen (2013) reported that pangolins in the Kalahari lost body condition during a winter and that some pangolins died of starvation.

Increased heterothermy in pangolins in response to food shortages in our study provides further evidence that the amplitude of the 24-h rhythm of body temperature can be used as an index of the physiological welfare of mammals. Biologging of body temperature is particularly useful for assessing the physiological welfare of pangolins, given that pangolins are difficult to monitor because they occur at low densities, are nocturnal, usually solitary and fossorial. Body temperature biologging, however, is invasive and difficult to implement widely. Our finding that there is a strong association between pangolin body temperature and food availability indicates that measuring food availability alone may be a useful surrogate for assessing the welfare of pangolins. We therefore propose that long-term monitoring of ant and termite availability, using pitfall traps as we did, or potentially through other methods, will be a useful tool in pangolin conservation, particularly given our finding that climate change in drylands likely is an additional threat to the survival of these threatened mammals.

In conclusion, pangolins regulated core body temperature within a narrow 24-h range when food resources were abundant but relaxed the precision of body temperature regulation when food resources became scarcer. In doing so, pangolins seemed to prioritize energy conservation over thermoregulation. Although this physiological flexibility allowed pangolins to cope with environmental temperatures and food resource fluctuations during our study, whether these adaptations may sufficiently buffer them against the increasing environmental temperatures and reduced food availability associated with climate change in drylands remains to be determined. We have shown that body temperature measurement using biologging, and insect abundance using pitfall trapping may be used as tools for understanding the physiological welfare of pangolins.

## Supplementary Material

Web_Material_coad068

## Data Availability

The data will be shared on reasonable request to the corresponding author.
