# Correction to: Temminck’s pangolins relax the precision of body temperature regulation when resources are scarce in a semi-arid environment

**DOI:** 10.1093/conphys/coad076

**Published:** 2023-09-12

*Conserv Physiol* 11(1): coad068; https://10.1093/conphys/coad068.

This is a correction to: Wendy Panaino and others, Temminck’s pangolins relax the precision of body temperature regulation when resources are scarce in a semi-arid environment, *Conservation Physiology*, Volume 11, Issue 1, 2023, coad068, https://doi.org/10.1093/conphys/coad068.

In the originally published version of this manuscript, the title incorrectly read ‘Temminck pangolins’ instead of ‘Temminck’s pangolins’.

This error has been corrected online.

