# Supplementary material for: Temminck’s pangolins relax the precision of body temperature regulation when resources are scarce in a semi-arid environment
Source: Conserv Physiol. 2023 Aug 28;11(1):coad068. doi: 10.1093/conphys/coad068 (PMC10465008; doi:10.1093/conphys/coad068)
Supplement: Web_Material_coad068 [file web_material_coad068.pdf]

SUPPLEMENTARY MATERIAL

Data logger implantation & removal

Timing

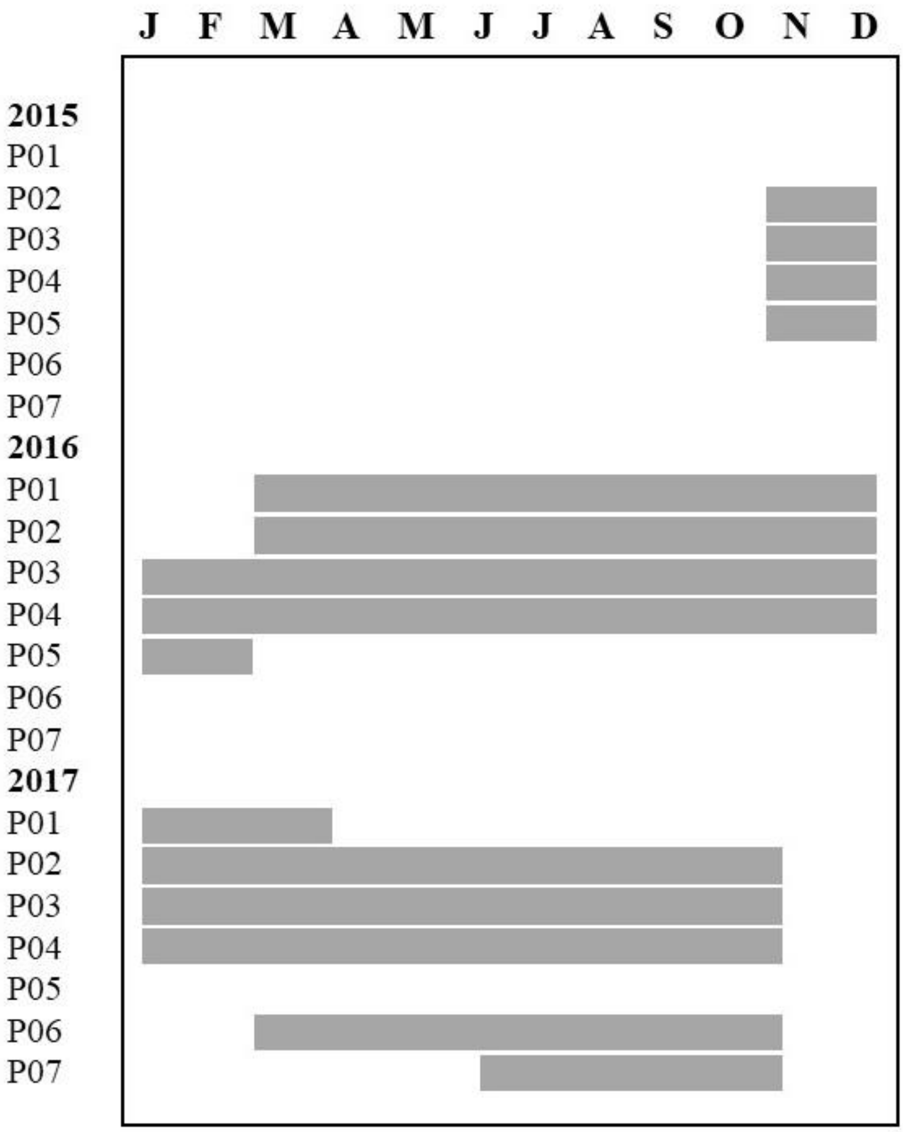

Figure S1. Start and end dates of implantation and removal of body temperature data loggers from seven pangolins between November 2015 to October 2017

Surgical procedures

Subcutaneous stitching of the *linea alba* was performed with 4/0 absorbable suture material (Viamac (USP), Scimitar Surgical Sutures, Gabler Medical (Pty) Ltd., Essex, UK), followed by 2/0 absorbable vicryl suture material (Viamac (USP), Scimitar Surgical Sutures, Gabler Medical (Pty) Ltd., Essex, UK) to close the skin. A non-steroidal anti-inflammatory meloxicam 0.5mg/kg (Metacam®, 5 mg/mlBoehringer Ingelheim Pharmaceuticals (Pty) Ltd., Johannesburg, South Africa) and a long-acting antibiotic (PeniLA, 0.1 ml/kg, penicillin, VIRBAC RSA (Pty) Ltd., Johannesburg, South Africa) were injected subcutaneously. Wound spray (Necrospray, oxytetracycline hydrochloride: 40 mg, gentian violet: 4 mg, Animal Health Division, Bayer HealthCare (Pty) Ltd., Kempton Park, South Africa) and tick grease (chlorfenvinphos: 0.3%, SWAVET RSA (Pty) Ltd., Johannesburg, South Africa) were applied to the wound site once suturing was completed.

Validation of pangolin activity

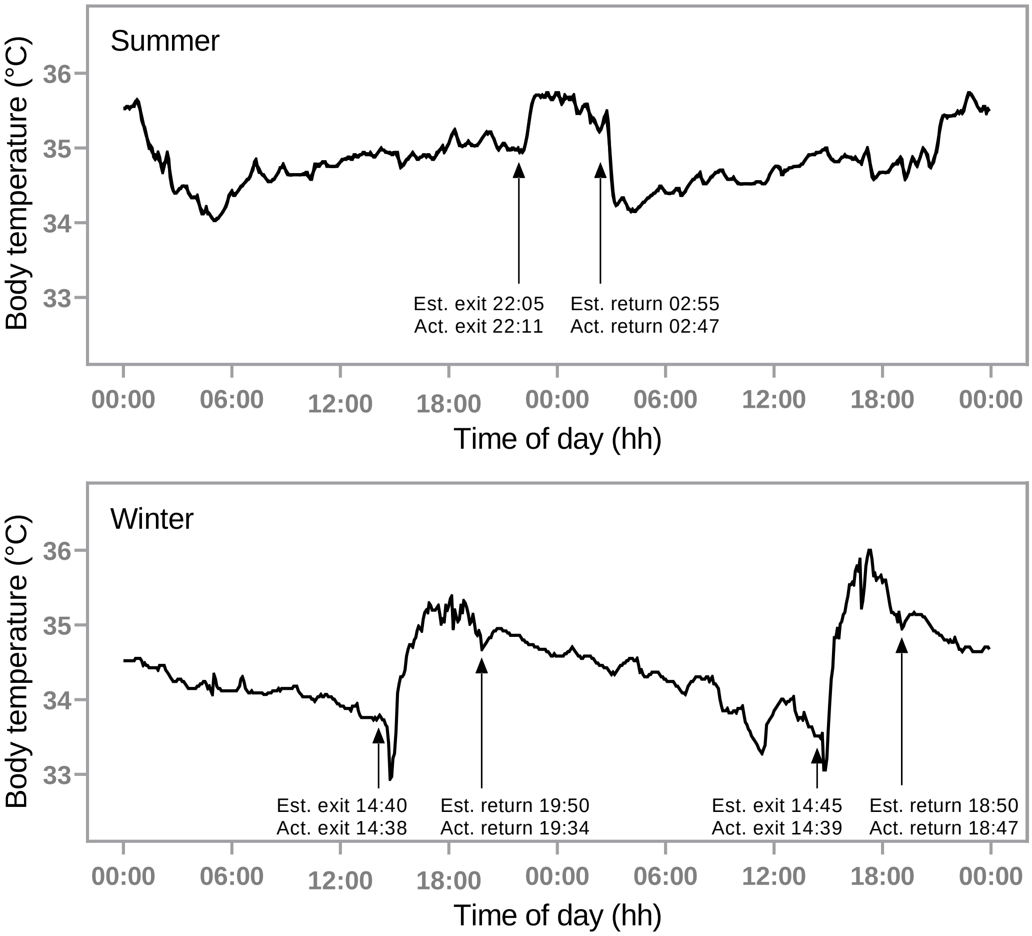

Figure S2. The 24h body temperature patterns of a pangolin, showing the estimated (Est.) and actual (Act.; camera trap data) burrow emergence and return times for two consecutive summer days (top) and two consecutive winter days (bottom)

Pangolin 24h body temperature records showed a notch (indicated by an increase or decrease in body temperature by at least 0.5°C in less than one hour; 93% of notches were ≥0.5°C for data points for which we had camera trap data for validation) around the times of emergence from and return to their burrows. We tested the accuracy of the body temperatures deviations to identify the time of emergence and return to the burrow by matching 178 camera trap times of emergence and 65 camera trap times of return with 24h body temperature records. The success of using of a conspicuous body temperature notch to detect burrow emergence or return was calculated by counting the number of times (reported as a percentage of time) the notch

was detectable to within one hour of the actual emergence and return using camera traps (Figure S2). The use of the body temperature notch to detect time of emergence (for when camera trap data were available) was successful for 89% of the time during autumn, for 89% of the time during spring, and for 100% of the time during winter. In other words, for only up to 11% of the time, depending on the season, the body temperature notch was not conspicuous enough to detect time of emergence from the burrow. During summer, however, the use of body temperature notches to detect time of emergence was only possible for 64% of the time because the notches were not as distinct during summer compared to the rest of the year. The low detection success during summer resulted in fewer estimated times of emergence being available for analysis during summer compared to the rest of the year. The use of the body temperature notch to detect time of return (for when camera trap data were available) was successful for 100% of the time during autumn, for 92% of the time during spring, for 100% of the time during winter, and for 93% of the time during summer. In other words, for only up to 8% of the time, depending on the season, the body temperature notch was not conspicuous enough to detect time of return to the burrow.

The time of emergence and return estimation error was determined by calculating the absolute difference between the estimated time of burrow emergence or return using 24h body temperature patterns and the actual time of burrow emergence or return using camera traps. On average, the time of emergence estimation error was 32 ± 28 (mean ± SD of total emergences) minutes for summer, 20 ± 22 minutes for autumn, 12 ± 11 minutes for winter, and 18 ± 15 minutes for spring. The time of return estimation error was 17 ± 18 (mean ± SD of total returns) minutes for summer, 13 ± 7 minutes for autumn, 11 ± 15 minutes for winter, and 21 ± 24 minutes for summer.

Frequency histograms created using activity obtained from camera traps only (n=243; 178 emergences, 65 returns) and activity derived from 24h body temperature only (n=4998; 2744 emergences, 2254 returns) revealed that the overall distribution of the data was similar for the two methods (Figure S3). We were therefore confident that 24h body temperature notches could be used to accurately estimate time of emergence and return to the burrow to within one hour of actual activity.

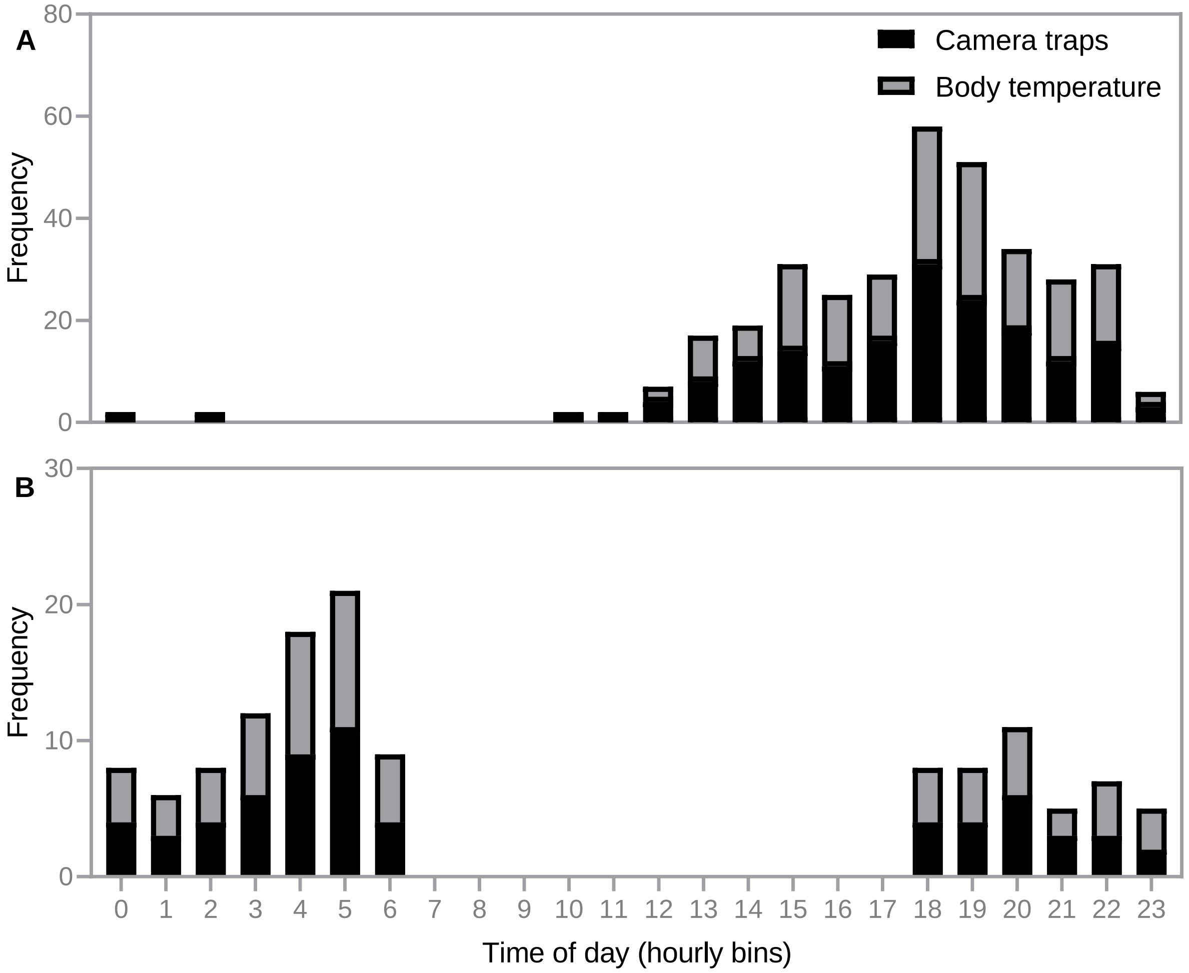

Figure S3. Frequency distributions of time of emergence (A) and return (B) data obtained directly from camera traps only (black bars) and indirectly from 24h body temperature patterns only (grey bars) for which camera trap data were available. The hourly bins represent the time of day during which emergence from or return to the burrow occurred (for example, 1 = 01h00-01h59 and 20 = 20h00-20h59)

Globe temperature

Raw data

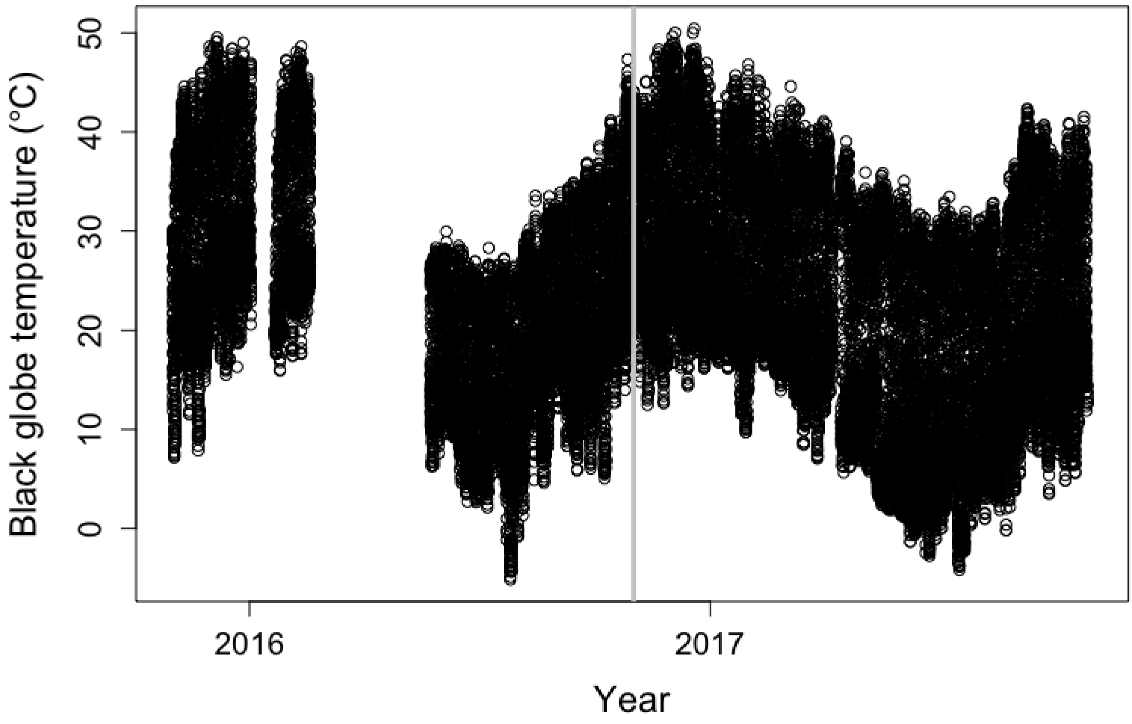

Figure S4. Raw black globe temperature recordings at the study site for the period 1 November 2015 to 31 October 2017. The gaps in the data occurred due to a failed weather station. The vertical grey line separates the data into two year-long periods: i) 1 November 2015 to 31 October 2016, ii) 1 November 2016 to 31 October 2017

Table S1. Days of data per season in each period of study

Period[1]	Season[2]	Days of data[3]
2015/2016	Summer	61
2015/2016	Autumn	8
2015/2016	Winter	92
2015/2016	Spring	61
2016/2017	Summer	90
2016/2017	Autumn	86
2016/2017	Winter	92
2016/2017	Spring	58

[1] 2015/2016: 1 November 2015 to 31 October 2016

2016/2017: 1 November 2016 to 31 October 2017

[2] Summer: 1 December to 28 February

Autumn: 1 March to 31 May

Winter: 1 June to 31 August

Spring: 1 September to 31 October (2-month duration because of split in Spring across periods)

[3] Missing days due to weather station failure

Emergence times

Raw data

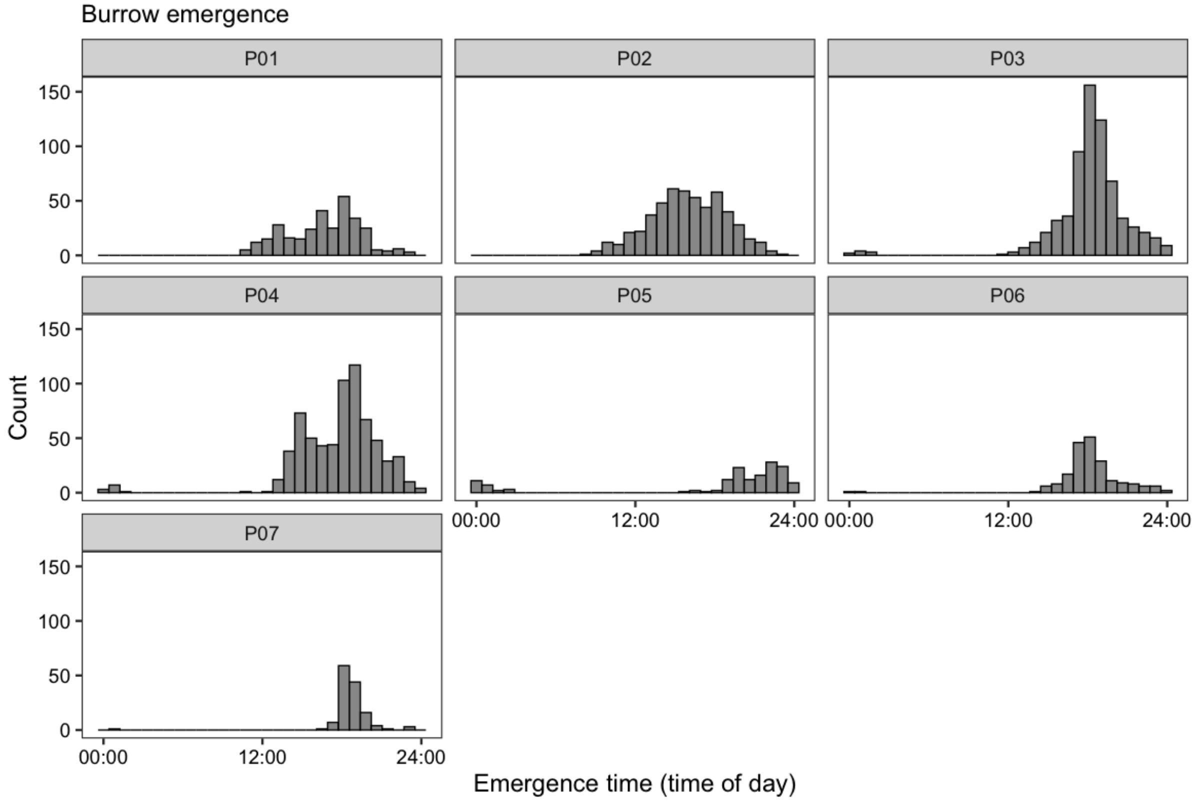

Figure S5. Distribution of burrow emergence times for each animal

Table S2. Percent emergence times between 00:00 and 05:00

Pangolin	Percent emergences				
	< 01:00	< 02:00	< 03:00	< 04:00	< 05:00
P01	0.00	0.00	0.00	0.00	0.00
P02	0.00	0.00	0.00	0.00	0.00
P03	0.90	1.34	1.34	1.34	1.34
P04	1.17	1.61	1.61	1.61	1.61

Pangolin	Percent emergences				
	< 01:00	< 02:00	< 03:00	< 04:00	< 05:00
P05	9.80	13.07	15.03	15.03	15.03
P06	0.99	0.99	0.99	0.99	0.99
P07	0.00	0.74	0.74	0.74	0.74

Daily minimum globe temperature

Table S3. Linear regression model of seasonal and yearly (period) differences in daily minimum globe temperature (ºC)

**Characteristic**	**Coefficient**	**95% CI**[1]
**(Intercept)**	22	21, 22
**Season**		
Summer	—	—
Spring	-7.2	-8.1, -6.4
Winter	-14	-15, -13
**Period**		
2015/2016	—	—
2016/2017	-4.0	-4.7, -3.3

[1] CI = Confidence Interval

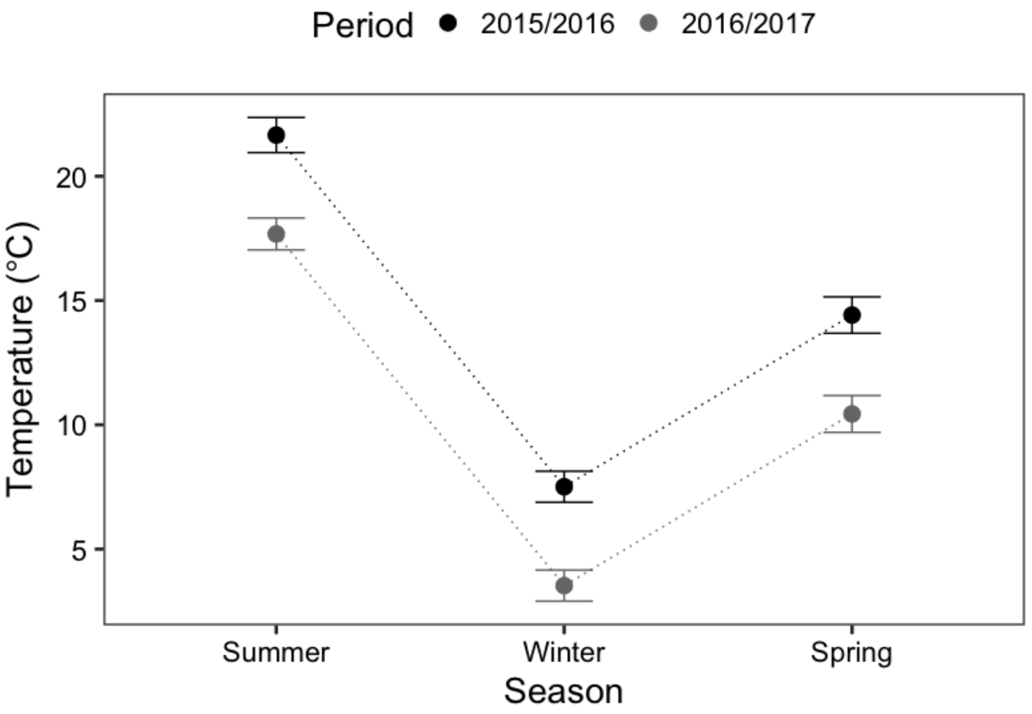

Figure S6. Estimate marginal means of daily minimum globe temperature by season, averaged over period. Error bars show the 95% confidence interval for the estimates

Table S4. Pairwise contrasts of estimated marginal means of daily minimum globe temperature

Contrast	Difference (ºC)	Standard error	p-value[1]
Summer - Spring	7.2	0.4	< 0.001
Summer - Winter	14.1	0.4	< 0.001
Spring - Winter	6.9	0.4	< 0.001
(2015/2016) - (2016/2017)	4.0	0.3	< 0.001

[1] P-value adjustment method: Tukey

Daily maximum globe temperature

Table S5. Linear regression model of seasonal and yearly (period) differences in daily maximum globe temperature (ºC)

Characteristic	Coefficient	95% CI[1]
**(Intercept)**	42	41, 43
**Season**		
Summer	—	—
Spring	-7.2	-8.3, -6.1
Winter	-15	-16, -14
**Period**		
2015/2016	—	—
2016/2017	0.71	-0.15, 1.6

[1] CI = Confidence Interval

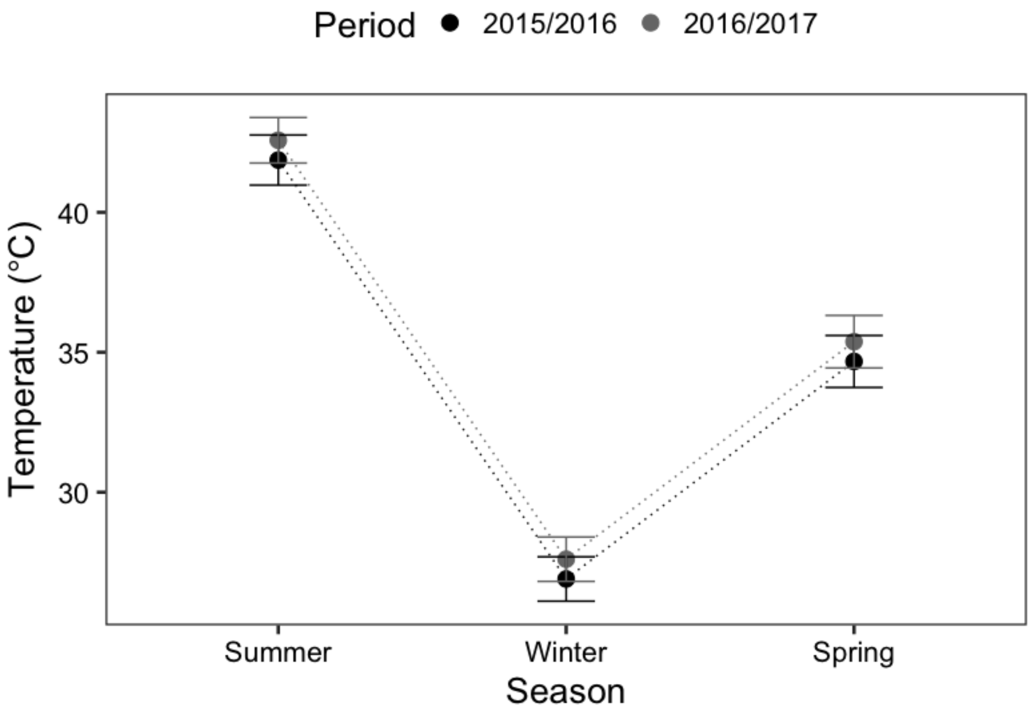

Figure S7. Estimate marginal means of daily maximum globe temperature by season, averaged over period. Error bars show the 95% confidence interval for the estimates

Table S6. Pairwise contrasts of estimated marginal means of daily maximum globe temperature

Contrast	Difference (ºC)	Standard error	p-value[1]
Summer - Spring	7.2	0.6	< 0.001
Summer - Winter	15.0	0.5	< 0.001
Spring - Winter	7.8	0.5	< 0.001
(2015/2016) - (2016/2017)	-0.7	0.4	0.105

[1] P-value adjustment method: Tukey

Daily amplitude of globe temperature

Table S7. Linear regression model of seasonal and yearly (period) differences in daily amplitude of globe temperature (ºC)

Characteristic	Coefficient	95% CI[1]
**(Intercept)**	20	19, 21
**Season**		
Summer	—	—
Spring	0.05	-1.2, 1.2
Winter	-0.82	-1.9, 0.26
**Period**		
2015/2016	—	—
2016/2017	4.7	3.8, 5.6

[1] CI = Confidence Interval

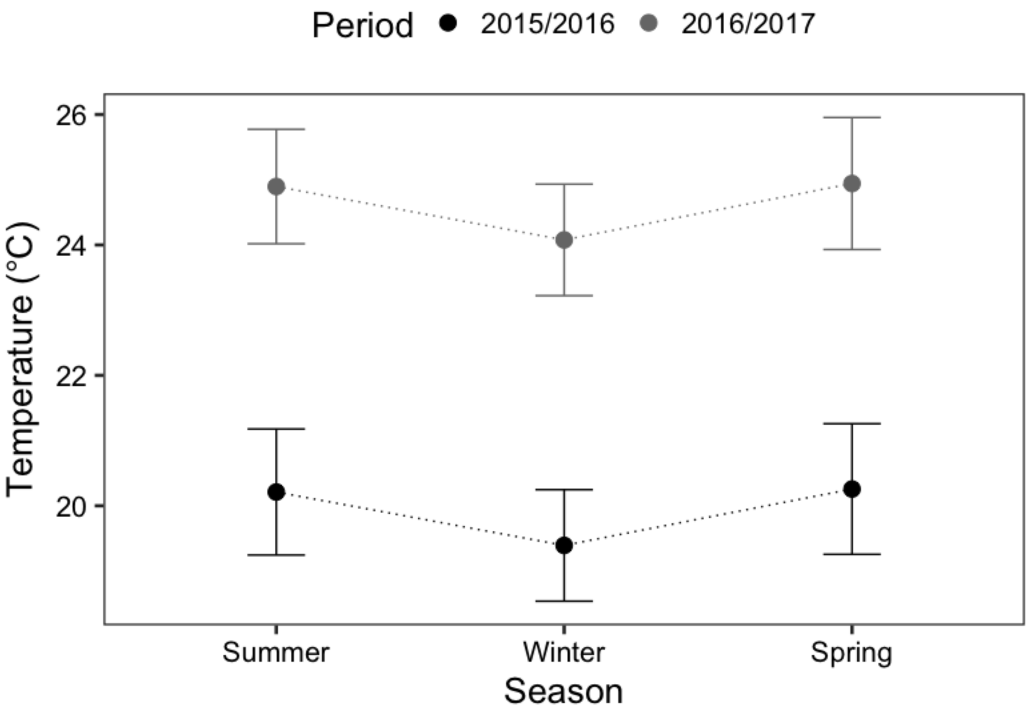

Figure S8. Estimate marginal means of daily amplitude of globe temperature by season, averaged over period. Error bars show the 95% confidence interval for the estimates.

.

Table S8. Pairwise contrasts of estimated marginal means of daily amplitude of globe temperature

**Contrast**	**Difference (ºC)**	**Standard error**	**p-value[1]**
Summer - Spring	0.0	0.6	0.997
Summer - Winter	0.8	0.5	0.294
Spring - Winter	0.9	0.6	0.302
(2015/2016) - (2016/2017)	-4.7	0.5	< 0.001

[1] P-value adjustment method: Tukey

Daily mean globe temperature

Table S9. Linear regression model of seasonal and yearly (period) differences in daily mean globe temperature (ºC)

**Characteristic**	**Coefficient**	**95% CI**[1]
**(Intercept)**	31	30, 32
**Season**		
Summer	—	—
Spring	-5.6	-6.5, -4.7
Winter	-15	-15, -14
**Period**		
2015/2016	—	—
2016/2017	-2.3	-2.9, -1.6

[1] CI = Confidence Interval

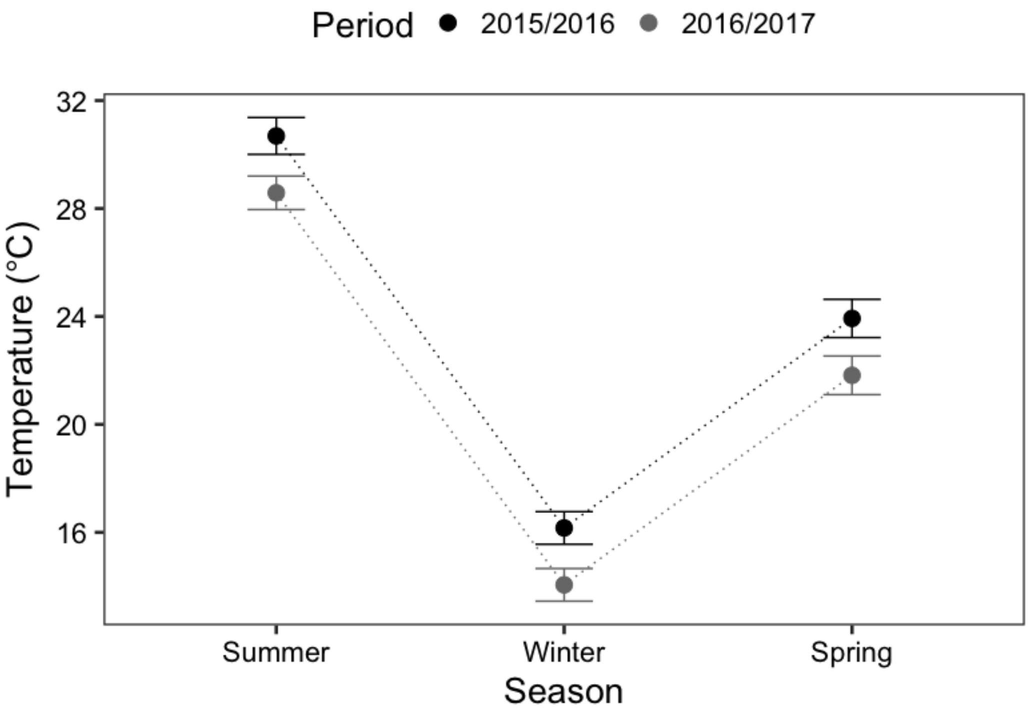

Figure S9. Estimate marginal means of daily mean globe temperature by season, averaged over period. Error bars show the 95% confidence interval for the estimates

Table S10. Pairwise contrasts of estimated marginal means of daily mean globe temperature

**Contrast**	**Difference (°C)**	**Standard error**	**p-value[1]**
Summer - Spring	6.8	0.4	< 0.001
Summer - Winter	14.5	0.4	< 0.001
Spring - Winter	7.8	0.4	< 0.001
(2015/2016) - (2016/2017)	2.1	0.3	< 0.001

[1] P-value adjustment method: Tukey

Rainfall

Raw data

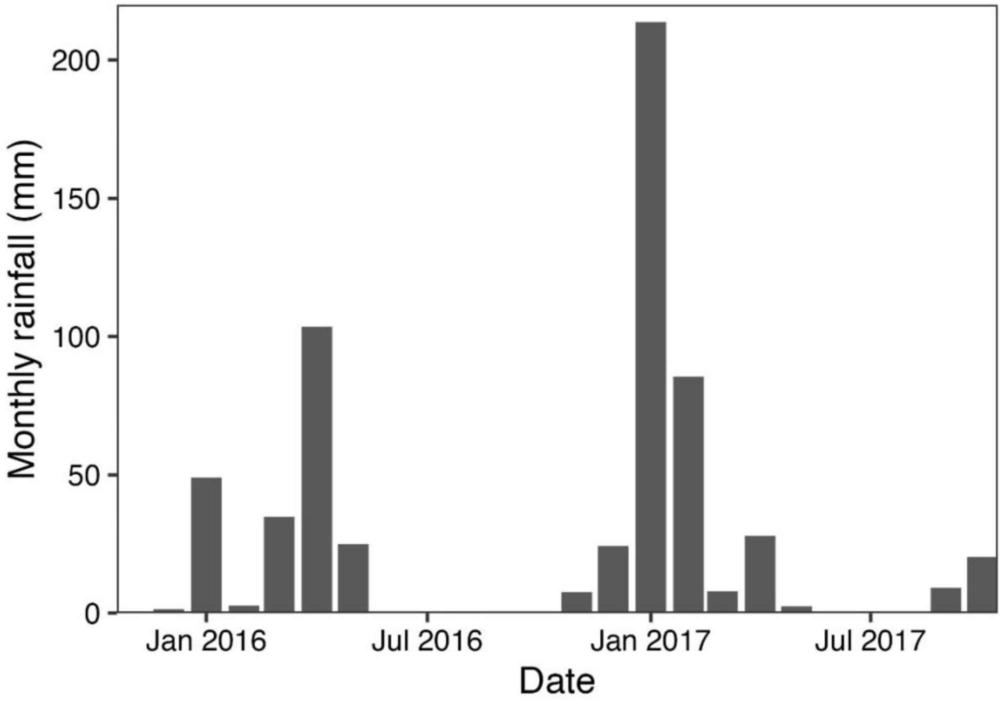

Figure S10. Raw monthly rainfall recordings at the study site for the period 1 November 2015 to 31 October 2017.

Seasons

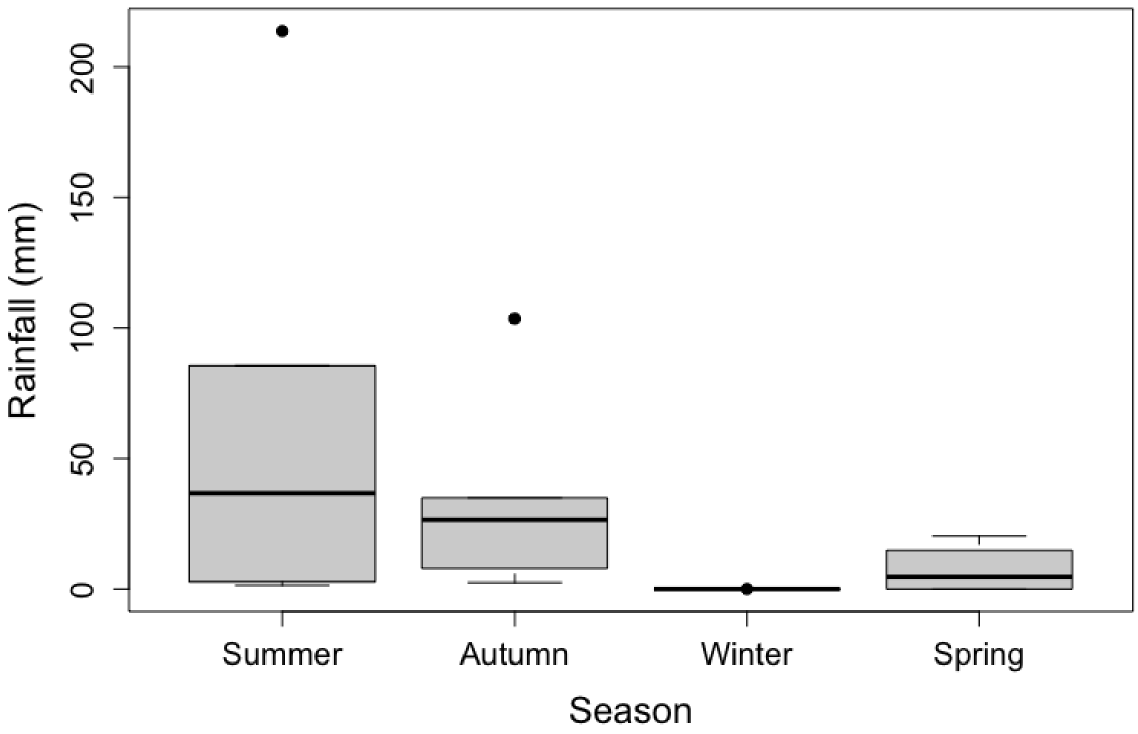

Figure S11. Tukey boxplot of seasonal rainfall across a two-year period (November 2015 to October 2017). Summer: 1 December to 28 February, Autumn: 1 March to 31 May, Winter: 1 June to 30 August, Spring: 1 September to 31 October

Table S11. Median monthly seasonal rainfall pattern across two years

**Characteristic**	**Summer**, N = 6[1]	**Autumn**, N = 6[1]	**Spring**, N = 4[1]	**Winter**, N = 6[1]
Rainfall (mm)	37 (8, 76)	26 (12, 33)	5 (0, 12)	0 (0, 0)

[1] Median (interquartile range)

Period

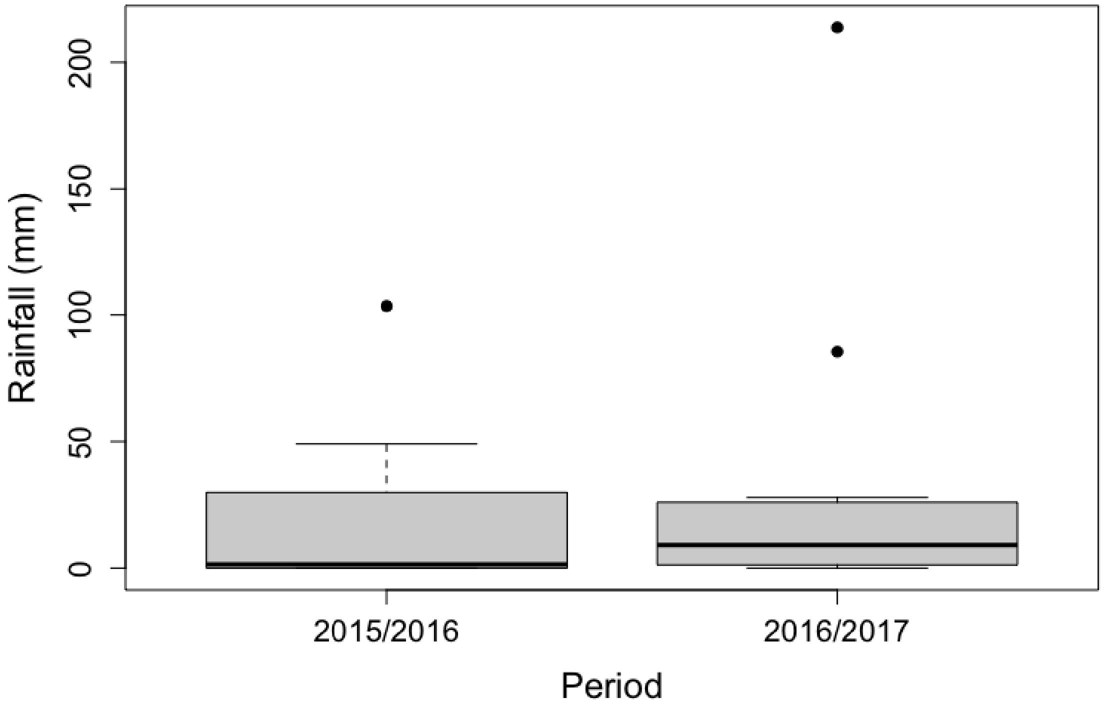

Figure S12. Tukey boxplot of monthly rainfall recordings at the study site for the two-year period from 1 November 2015 to 31 October 2017. 2015/2016 period: 1 November 2015 to 31 October 2016, 2016/2017 period: 1 November 2016 to 31 October 2017

Table S12. Median and mean monthly rainfall, and total annual rainfall

**Characteristic**	**2015/2016**, N = 11	**2016/2017**, N = 11
Rainfall (mm)		
Median (IQR)[1]	1 (0, 30)	9 (1, 26)
Mean (SD)[2]	20 (33)	36 (64)
Total	217	391

[1] IQR: interquartile range

[2] SD: standard deviation

Prey abundance

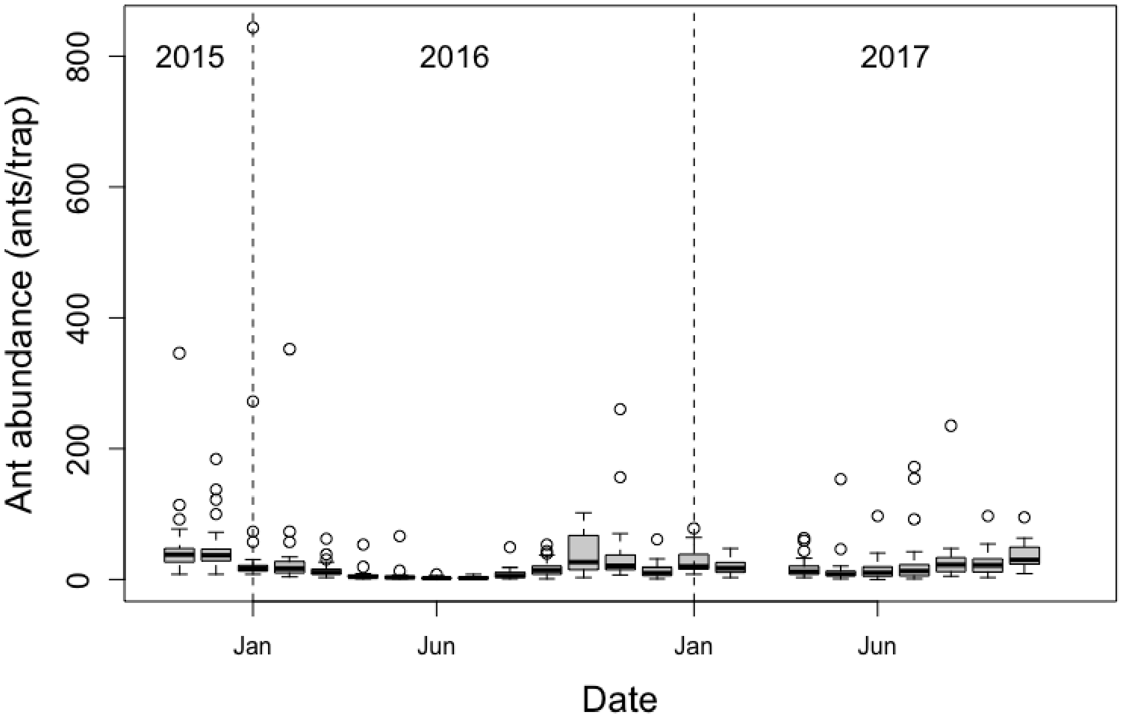

Figure S13. Median (interquartile range, minimum, and maximum) monthly ant abundance for the 24-month study period (November 2015 to October 2017). The data are aggregated across 30 transects. Data for March 2017 are missing as transects were not conducted during that month

Table S13. Generalised linear mixed-effects model (negative binomial link function) results of the interannual and seasonal differences in ant abundance

Characteristic	Incidence rate ratio	95% CI[1]
**(Intercept)**	18.8	14.6, 24.2
**Season**		
Summer	—	—
Autumn	0.39	0.30, 0.51
Spring	1.07	0.83, 1.39
Winter	0.33	0.25, 0.43
**Period**		
2015/2016	—	—
2016/2017	1.78	1.46, 2.17

[1] CI = Confidence Interval

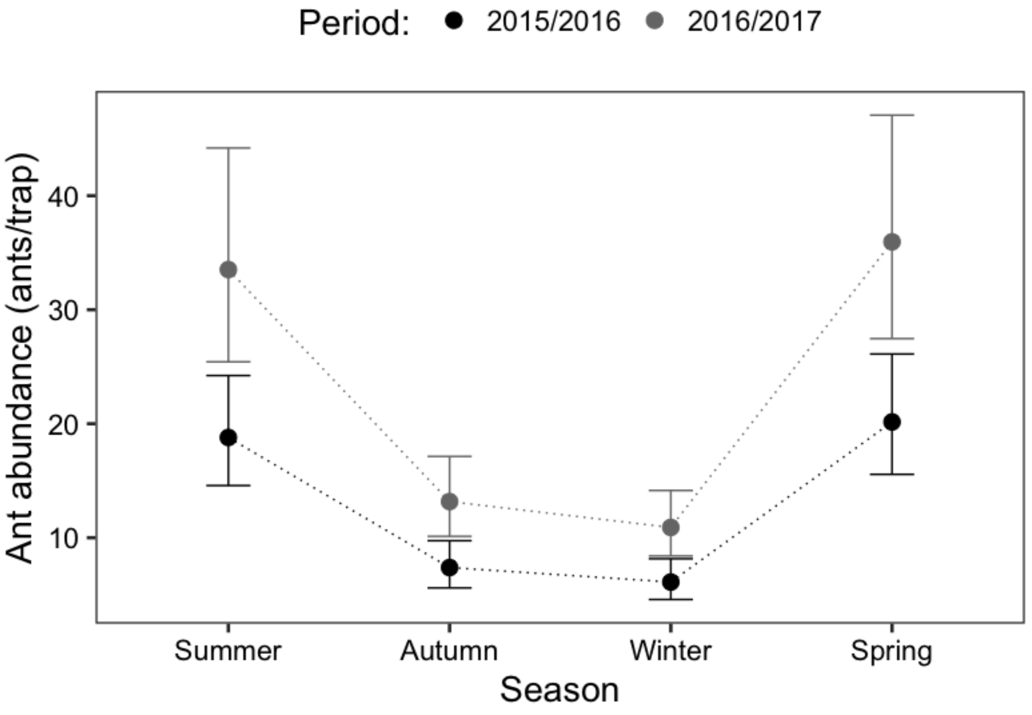

Figure S14. Estimated marginal means of prey abundance by season, averaged over period. Error bars show the 95% confidence interval for the estimates. Values are back-transformed from the log scale

Table S14. Pairwise contrasts of estimated marginal means for prey abundance (ants/trap)

**Contrast**	**Ratio**	**Standard error**	**p-value**
Summer - Autumn	2.55	0.35	< 0.001
Summer - Spring	0.93	0.12	0.953
Summer - Winter	3.07	0.44	< 0.001
Autumn - Spring	0.37	0.05	< 0.001
Autumn - Winter	1.21	0.17	0.531
Spring - Winter	3.30	0.46	< 0.001
(2015/2016) - (2016/2017)	0.56	0.06	< 0.001

Tests are performed on the log scale

P-value adjustment method: Tukey

Null ratio = 1

Body temperature

Daily minimum body temperature

Table S15. Linear mixed-effects model results of the interannual and seasonal differences in daily minimum body temperature (°C)

Characteristic	Coefficient	95% CI[1]
**(Intercept)**	34	33, 34
**Season**		
Summer	—	—
Autumn	-0.36	-0.43, -0.29
Spring	-0.16	-0.24, -0.09
Winter	-0.73	-0.80, -0.67
**Period**		
2015/2016	—	—
2016/2017	0.35	0.30, 0.40

[1] CI = Confidence Interval

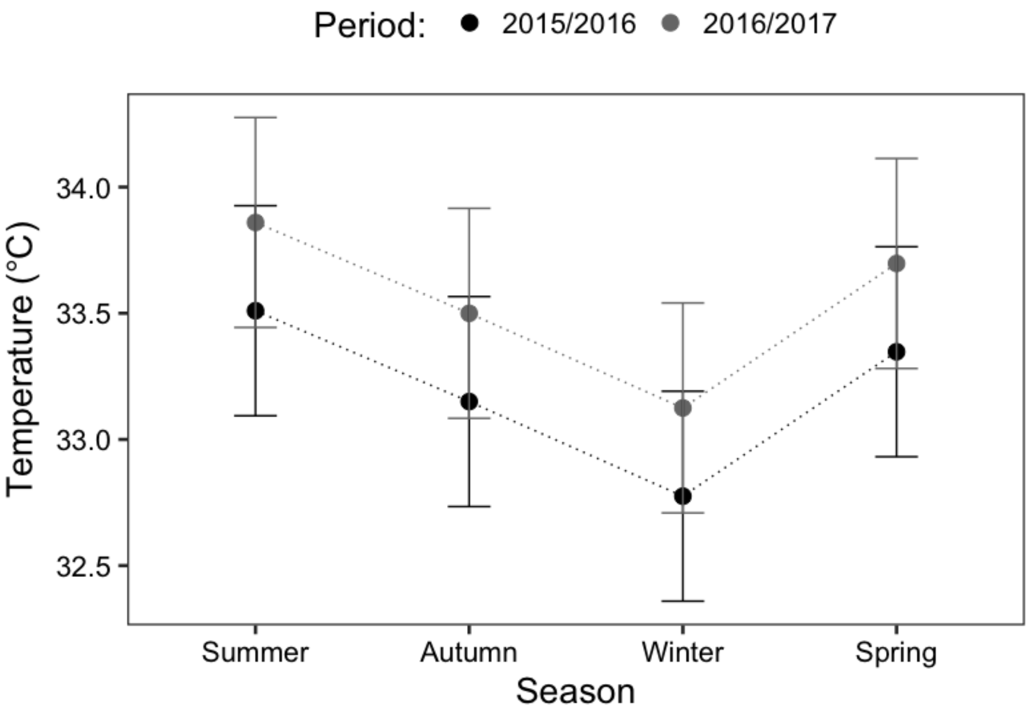

Figure S15. Estimate marginal means of daily minimum body temperature by season, averaged over period. Error bars show the 95% confidence interval for the estimates

Table S16. Pairwise contrasts of estimated marginal means for minimum body temperature

Contrast	Difference (°C)	Standard error	p-value[1]
Summer - Autumn	0.36	0.04	< 0.001
Summer - Spring	0.16	0.04	< 0.001
Summer - Winter	0.73	0.04	< 0.001
Autumn - Spring	-0.20	0.04	< 0.001
Autumn - Winter	0.37	0.03	< 0.001
Spring - Winter	0.57	0.03	< 0.001
(2015/2016) - (2016/2017)	-0.35	0.03	< 0.001

**Contrast**	**Difference (ºC)**	**Standard error**	**p-value**[1]

[1] P-value adjustment method: Tukey

Daily maximum body temperature

Table S17. Linear mixed-effects model results of the interannual and seasonal differences in daily maximum body temperature (ºC)

**Characteristic**	**Coefficient**	**95% CI**[1]
**(Intercept)**	36	35, 36
**Season**		
Summer	—	—
Autumn	-0.02	-0.07, 0.03
Spring	0.06	0.01, 0.12
Winter	0.07	0.02, 0.11
**Period**		
2015/2016	—	—
2016/2017	-0.27	-0.30, -0.23

[1] CI = Confidence Interval

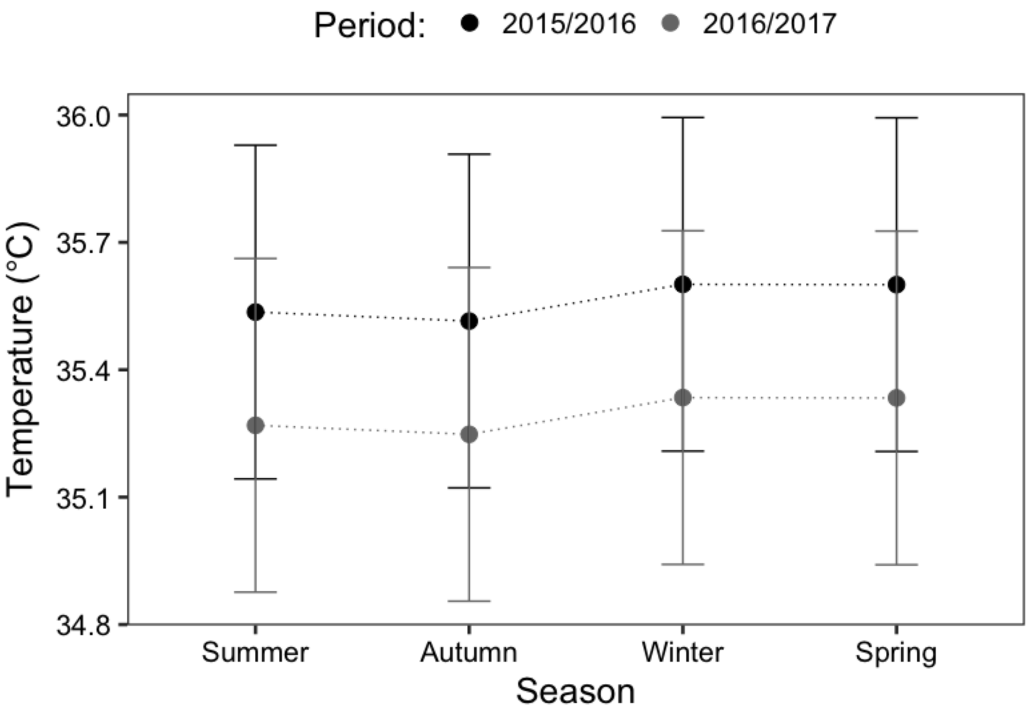

Figure S16. Estimate marginal means of daily maximum body temperature by season, averaged over period. Error bars show the 95% confidence interval for the estimates

Table S18. Pairwise contrasts of estimated marginal means for maximum body temperature

**Contrast**	**Difference (°C)**	**Standard error**	**p-value[1]**
Summer - Autumn	0.02	0.02	0.807
Summer - Spring	-0.06	0.03	0.061
Summer - Winter	-0.07	0.02	0.03
Autumn - Spring	-0.09	0.02	0.002
Autumn - Winter	-0.09	0.02	< 0.001
Spring - Winter	0.00	0.02	> 0.9
(2015/2016) - (2016/2017)	0.27	0.02	< 0.001

Contrast	Difference (ºC)	Standard error	p-value[1]

[1] P-value adjustment method: Tukey

Daily amplitude of body temperature

Table S19. Linear mixed-effects model results of the interannual and seasonal differences for daily amplitude of body temperature (ºC)

Characteristic	Coefficient	95% CI[1]
**(Intercept)**	2.0	1.8, 2.3
**Season**		
Summer	—	—
Autumn	0.34	0.26, 0.42
Spring	0.23	0.14, 0.32
Winter	0.80	0.72, 0.88
**Period**		
2015/2016	—	—
2016/2017	-0.62	-0.68, -0.55

[1] CI = Confidence Interval

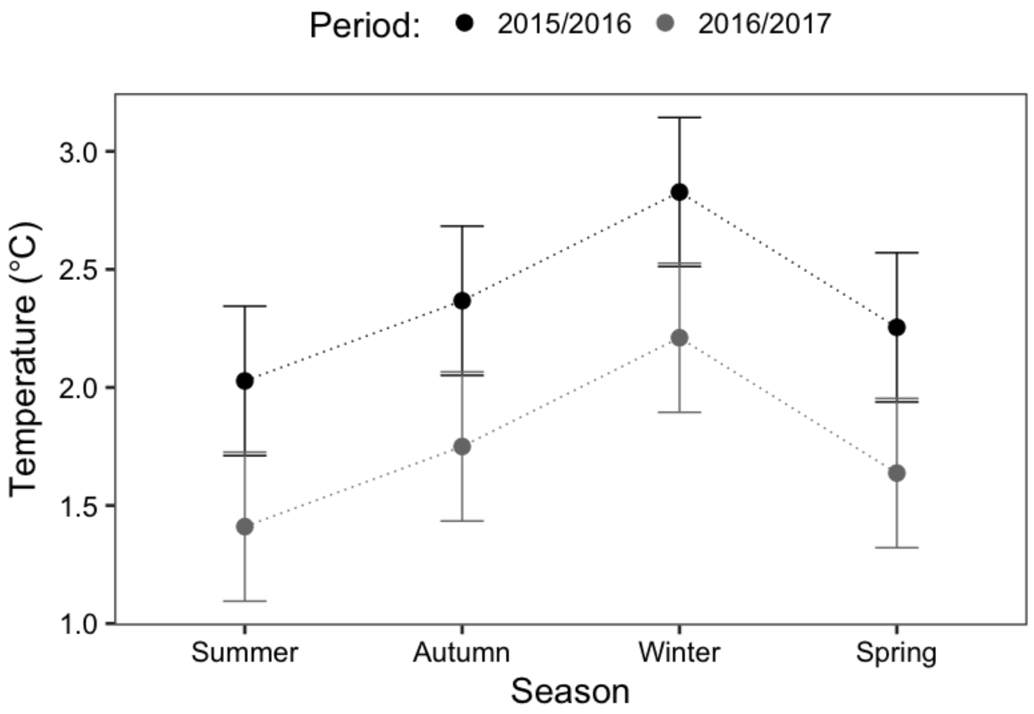

Figure S17. Estimate marginal means for daily amplitude of body temperature by season, averaged over period. Error bars show the 95% confidence interval for the estimates

Table S20. Pairwise contrasts of estimated marginal means for daily amplitude of body temperature

Contrast	Difference (ºC)	Standard error	p-value[1]
Summer - Autumn	-0.34	0.04	< 0.001
Summer - Spring	-0.23	0.05	< 0.001
Summer - Winter	-0.80	0.04	< 0.001
Autumn - Spring	0.11	0.04	0.048
Autumn - Winter	-0.46	0.04	< 0.001
Spring - Winter	-0.57	0.04	< 0.001
(2015/2016) - (2016/2017)	0.62	0.03	< 0.001

Contrast	Difference (ºC)	Standard error	p-value[1]

[1] P-value adjustment method: Tukey

Daily mean body temperature

Table S21. Linear mixed-effects model results of the interannual and seasonal differences in daily mean body temperature (ºC)

Characteristic	Coefficient	95% CI[1]
**(Intercept)**	35	34, 35
**Season**		
Summer	—	—
Autumn	-0.25	-0.28, -0.22
Spring	-0.26	-0.29, -0.23
Winter	-0.34	-0.37, -0.32
**Period**		
2015/2016	—	—
2016/2017	0.00	-0.02, 0.02

[1] CI = Confidence Interval

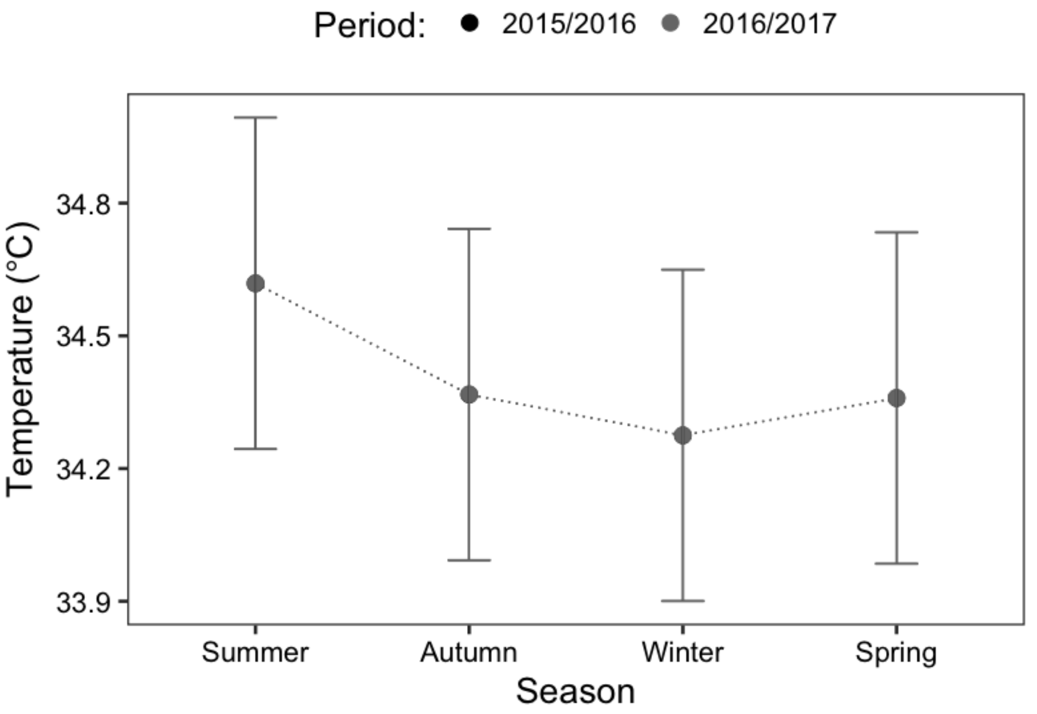

Figure S18. Estimate marginal means of daily mean body temperature by season, averaged over period. Error bars show the 95% confidence interval for the estimates

Table S22. Pairwise contrasts of estimated marginal means for daily mean body temperature

Contrast	Difference (°C)	Standard error	p-value[1]
Summer - Autumn	0.25	0.01	< 0.001
Summer - Spring	0.26	0.02	< 0.001
Summer - Winter	0.34	0.01	< 0.001
Autumn - Spring	0.01	0.01	> 0.9
Autumn - Winter	0.09	0.01	< 0.001
Spring - Winter	0.08	0.01	< 0.001
(2015/2016) - (2016/2017)	0.00	0.01	> 0.9

Contrast	Difference (°C)	Standard error	p-value[1]

[1] P-value adjustment method: Tukey
